# Randomised controlled trial of the effect, cost and acceptability of a bronchiectasis self-management intervention

**DOI:** 10.1177/1479973120948077

**Published:** 2020-12-02

**Authors:** Claire Brockwell, Andrea Stockl, Allan Clark, Garry Barton, Mark Pasteur, Robert Fleetcroft, Janice Hill, Andrew M Wilson

**Affiliations:** 1 Norwich Medical School, 6106 University of East Anglia, Norwich, UK; 2 Department of Respiratory Medicine, 6107 Norfolk and Norwich University Hospital NHS Foundation Trust, Colney Lane, Norwich, UK; 3 Acle Medical Partnership, Bridewell Lane, Acle, Norfolk, UK; 4 232261 Norfolk Community Health and Care NHS Trust, Elliot House, Norwich, Norfolk, UK

**Keywords:** Bronchiectasis, mixed-methods, patient self-management plans, self-efficacy to manage chronic disease scale, ST George’s respiratory questionnaire

## Abstract

**Background:**

Patient self-management plans (PSMP) are advised for bronchiectasis but their efficacy is not established. We aimed to determine whether, in people with bronchiectasis, the use of our bronchiectasis PSMP – Bronchiectasis Empowerment Tool (BET), compared to standard care, would improve self-efficacy.

**Methods:**

In a multi-centre mixed-methods randomised controlled parallel study, 220 patients with bronchiectasis were randomised to receive standard care with or without the addition of our BET plus education sessions explaining its use. BET comprised an action plan, indicating when to seek medical help based on pictorial represented indications for antibiotic therapy, and four educational support sections. At baseline and after 12 months, patients completed the Self-Efficacy to Manage Chronic Disease Scale (SEMCD), St George’s Respiratory Questionnaire (SGRQ), EQ-5D-3 L (to calculate Quality Adjusted Life Years (QALYs) and cost questionnaires. Qualitative data were obtained by focus groups.

**Results:**

The recruitment to the study was high (63% of eligible patients agreeing to participate) however completion rate was low (57%). BET had no effect on SEMCD (mean difference (0.14 (95% confidence interval (95%CI) −0.37 to 0.64), p = 0.59) or SGRQ, exacerbation rates, overall cost to the NHS or QALYs. Most had developed their own techniques for monitoring their condition and they did not find BET useful as it was difficult to complete. Participant knowledge was good in both groups.

**Conclusion:**

The demand for patient support in bronchiectasis was high suggesting a clinical need. However, the BET did not improve self-efficacy, health related quality of life, costs or clinically relevant outcome measures. BET needs to be modified to be less onerous for users and implemented within a wider package of care. Further studies, particularly those evaluating people newly diagnosed with bronchiectasis, are required and should allow for 50% withdrawal rate or utilise less burdensome outcome measures.

**Clinical trials registration:**

ISRCTN ISRCTN 18400127. Registered 24 June 2015. Retrospectively Registered

## Background

Bronchiectasis, a chronic lung disease characterised by chronic purulent sputum production, breathlessness and cough, is managed with airway clearance techniques, airway pharmacotherapy and appropriate use of antibiotics, along with patient education and disease monitoring.^1^ People with bronchiectasis often have impaired health related quality of life (HRQOL)^2^; and can experience repeated exacerbations due to lung infection resulting in deterioration in symptoms and increased hospital bed days and cost.^3^

Living with bronchiectasis results in considerable burden for patients, therefore methods of improving patient centred care are required to improve patient empowerment.^4^ Patient Self-Management Plans (PSMP) aim to do this and have been shown to improve health outcomes for adults with asthma^5^ and to be cost-effective.^6^ Indeed the recent European Multicentre Bronchiectasis Audit and Research Collaboration (EMBARC) consensus statement about research prorities highlighted the need for studies to determine the effectiveness of PSMP in bronchiectasis.^7^ A recently published systematic review concluded that there was insufficient evidence to determine whether self-management interventions are beneficial for people with bronchiectasis.^8^

We developed a self-management intervention for bronchiectasis (the Bronchiectasis Empowerment Tool (BET)) which was based on British Thoracic Society Guidelines, patient consultation and available literature on the patient perspective and needs for bronchiectasis self-management.^9^ It contained a 1 page action plan (which advises on actions depending on different circumstances) consisting of 3 action points, as is recommended,^10^ embedded in a document with written information and was supported by one to one education.

The study aimed to test whether, in people with bronchiectasis, the use of BET, compared to standard care, would improve self-efficacy using the Self-Efficacy to Manage Chronic Disease Scale (SEMCD),^11^ as this is a fundamental aspect of self-managment.^12^ Secondary aims were to assess the effect of BET on HRQOL and disease-related knowledge and to determine whether it was cost effective. We also aimed to explore the participants’ acceptability of BET.

## Methods

### Design

This was a multi-centre parallel randomised controlled mixed-methods parallel study of BET in people with bronchiectasis over a 12 month period. Participants from six hospitals (one bronchiectasis specialist centre, four local hospitals with specialist respiratory nursing support and one community hospital) in East Anglia, UK were recruited from May 2013 to April 2015. The study was conducted in accordance with Good Clinical Practice and all participants gave written informed consent. It had ethical approval (13/SC/0140) and was registered on a trials database (ISRCTN 18400127).

### Participants

Patients, of either gender, were included if they were older than 18 years, had a diagnosis of bronchiectasis confirmed on high resolution computed tomography (HRCT) and at least one exacerbation within the previous 12 months requiring treatment with antibiotics. Patients with cystic fibrosis or traction bronchiectasis, severe or uncontrolled co-morbid disease, impairment in cognitive functioning or did not speak English language were excluded. Patients currently using a written patient self-management plan or involved in the design of BET were also excluded.

### Randomisation

Eligible participants were randomised to the intervention or control groups, after completion of the baseline assessments, on a 1:1 basis using a computer generated code created by the study statistician with stratification according to hospital centre and severity of disease (four or more exacerbations in the last 12 months versus less than four) with code concealment in sequential opaque envelopes. Treatment allocation was undertaken by an unblinded researcher. All eligible participants received the contemporaneous British Lung Foundation Bronchiectasis Patient Information Sheet and Bronchiectasis Patient Information Leaflet from the British Thoracic Society/Association of Chartered Physiotherapists Respiratory Care Guidelines.^13^

### Intervention

Participants randomised to the intervention group received the BET document plus education sessions about its use. BET is a 48 page A5 booklet and comprises an action plan, four educational support sections each with notepads to assist in keeping track of their health, and links to on-line resources. The action plan is based on the indications for antibiotic therapy from the BTS bronchiectasis guidelines (sputum purulence, sputum volume and cough/wheeze/breathlessness) and pictorially represents easily recognisable health changes indicating when to seek medical help, to minimise barriers of health literacy. The educational support sections comprise information about general health, sputum clearance techniques and medication. There is a section for recording each course of antibiotic and date of sputum microbiology.

An un-blinded researcher (CB), previously a respiratory nurse, provided education about BET via four brief telephone conversations (lasting on average 10, 7, 5 and 2 minutes) delivered on consecutive days at the beginning of the study; these covered the use of the action plan and the information, monitoring and reference sections. Participants were given the opportunity to ask questions and to practice using the tool. Patients were provided with a contact number for information about the study and use of BET (but not for clinical queries). Participants’ healthcare providers were provided with brief information about BET in a letter.

### Control

Participants within the control group received standard care whereby patients attended routine appointment and were guided on their management according to current practice as per the BTS bronchiectasis guidelines.

### Measurements

Patients received the six item SEMCD to assess self-efficacy as it is a valid, responsive tool with high internal consistency in chronic disease, ranging between 1 and 10 with 10 scoring total confidence in managing disease^11^ and used to evaluate self-management programmes^14^; the St George’s Respiratory Questionnaire (SGRQ)^15^ to assess disease HRQOL as it has been validated for use in bronchiectasis^16^; the EuroQol-5D 3 level version (EQ-5D-3 L)^17^ to assess HRQOL; and cost questionnaires at baseline and every 3 months by post in a reply paid envelope. The Lung Information Needs Questionnaire (LINQ),^18^ which assesses knowledge and behaviour is validated in patients with chronic obstructive pulmonary disease but is easily transferable to bronchiectasis was completed at baseline and after 12 months. As no appropriate validated questionnaire existed which addressed the participants’ knowledge and confidence about bronchiectasis a new questionnaire was created in consultation with the research team and lay advisors was completed after12 months by participants. Patients who failed to return the questionnaires were sent a reminder questionnaire by post. The number of exacerbations of bronchiectasis,^19^ medical contacts and sputum microbiology requests were obtained from cost questionnaires and hospital records.

Two focus groups, comprising 4 participants each, purposively sampled to include patients with mild and severe disease from the intervention group, were facilitated by CB under supervision of AS (qualitative research expert) using a semi-structured interviewing technique, to explore participants’ perceptions of BET.

### Analysis

The primary outcome was the change from baseline in SEMCD. A sample size of 154 patients has 80% power to detect a treatment difference (two sided 5% significance) of 1 unit (10% of maximum score) of the SEMCD with a standard deviation of 2.2 units.^20^ We expected a withdrawal of 30% based study in chronic obstructive pulmonary disease with similar questionnaire burden,^21^ and therefore 220 patients were entered into the study. All data were double entered and discrepancies resolved by re-examining the source data. LINQ was analysed using the LINQ Scoring Tool (www.linq.org.uk). The Bronchiectasis Aetiology Comorbidity Index was calculated from clinical data.^22^

The analysis was based on an intention-to-treat approach. Change from baseline for primary and secondary endpoints was compared between groups using a general linear model adjusted for baseline severity. Total exacerbations and unscheduled care were both compared using negative binomial regression and reported as the incidence rate ratio which is the ratio of the event rates between the study arms. Adjusted analyses were conducted by additionally including the baseline value in the model as a covariate, e.g. for the SEMCD outcome we adjusted for the baseline measure of SEMCD. Data are presented as mean and standard deviation. The analysis was undertaken using Stata 16.1/SE.

Recordings of the focus groups were transcribed and a review of the data generated initial codes. Data from the focus groups were analysed in parallel to increase rigour.^23^ We used Microsoft Office Excel and computer assisted qualitative data analysis software (Nvivo11) to perform an inductive thematic analysis where patterns and clusters of linked data were organised into themes.^24,25^ In the results section we show selected quotes to illustrate the participants’ experience of using BET.

### Economic evaluation

Costs were estimated from the perspective of the NHS. The intervention costs were for a specialist nurse to arrange and conduct telephone education sessions, who would require 2 hour 1:1 training, and BET booklet printing. In the cost questionnaires, participants reported both hospital and community health visits. Unit costs were assigned to all items of resource use (£GBP ($USD) for the 2014–15 financial year).^26,27^

Responses to the EQ-5D-3 L were converted into utility scores^28^ using the UK York A1 tariff.^29^ Quality Adjusted Life Year (QALY) scores were subsequently calculated using the area under the curve approach.^30^ Multiple imputation was performed to account for missing cost and outcome data.^31^ Regression analysis^32^ was subsequently used to estimate the mean incremental cost (mean difference in cost) and effect (QALY gain) between the two groups and the incremental cost-effectiveness ratio (ICER).^33^ The cost effectiveness acceptability curve (CEAC), which estimates the probability of the intervention being cost-effective,^34^ was estimated at a value of £20,000 ($26,400) per QALY.

## Results

**Figure 1.**
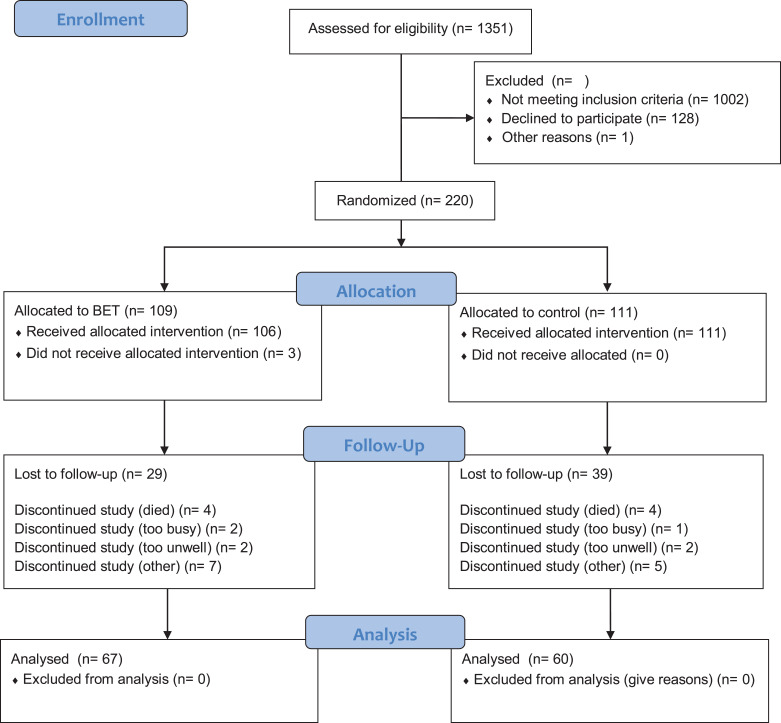
Disposition of patients.

The intention-to-treat analysis included 220 randomised patients, of which 155 (69%) were female, which represented 63.2% of eligible individuals (Figure 1). They had a mean (standard deviation) age of 66.9 (12.0) years, FEV1 1.84 (0.69) L, SEMCD 7.02 (2.0), total SGRQ 42.4 (19.1) and a median (inter quartile range) time from diagnosis of 5 years.^2–14^ The two groups were well balanced at baseline and hence no adjustment to the analysis was required to account for baseline factors (Table 1). The withdrawal rate was higher than expected with only 127 individuals (57%) returning the primary outcome questionnaire at 12 months. There was no difference in the change in SEMCD between the two study arms. The data were very slightly negatively skewed, but re-analysis using the bootstrap with 1,000 iterations gave similar results particularly for the adjusted analysis (unadjusted p = 0.96, adjusted p = 0.60) so that the results are not sensitive to the violation of the assumptions of the t-test. There were no significant differences between intervention and control for change in SGRQ, exacerbation rate, LINQ score or sputum microbiology requests (Table 2). In addition there were no differences between the intervention and control at any of the 3 month time points for any of the variables. Both groups were confident in managing their condition at the end of the study (Table 3).

**Table 1.** Summary of baseline characteristics for all individuals.

Factor	Control	Intervention
Age (years)	66.3 (13.4)	67.4 (10.5)
Gender		
f	78 (70.3%)	73(67.0%)
m	33 (29.7%)	36 (33.0%)
Smoking status		
Current smoker	7 (6.5%)	1 (1.0%)
Ex-smoker	45 (41.7%)	47 (44.8%)
Never smoked	56 (51.9%)	57 (54.3%)
FEV1 (L)	1.9 (0.8)	1.8 (0.6)
%,	82.3 (25.5)	75.4 (21.7)
FVC (L)	2.8 (0.9)	2.8 (0.9)
%,	96.4 (20.9)	91.0 (21.5)
Exacerbations		
>=4/year	40 (36.0%)	41 (37.6%)
<4/year	71 (64.0%)	68 (62.4%)
Exacerbations,	2.6 (3.0)	3.1 (3.0)
SEMCD score,	6.8 (2.1)	7.2 (1.9)
SGRQ total,	42.7 (21.1)	42.1 (17.0)
SGRQ symptoms,	55.9 (25.3)	57.1 (23.2)
SGRQ activity,	50.1 (27.8)	50.0 (23.0)
SGRQ impact,	34.2 (19.9)	32.1 (15.8)
Microbiology data (year before consent)		
Pseudomonas organism		
None	64 (72.73)	70 (77.78)
One	12 (13.64)	11 (12.22)
Two or more	12 (13.64)	9 (10.00)
Haemophilus organism		
None	77 (87.50)	75 (83.33)
One	7 (7.95)	6 (6.67)
Two or more	4 (4.55)	9 (10.00)
BACI score		
None or one	72 (64.9)	50 (45.9)
Two or three	21 (18.9)	37 (33.9)
Four or more	18 (16.2)	22 (20.2)
Median, IQR	0 (0–3)	3 (0–3)
LINQ score,	12.75 (2.50)	12.58 (2.40)
Disease knowledge	3.04 (0.73)	2.99 (0.70)
Medicines	2.34 (0.67)	2.37 (0.66)
Self-management	3.51 (1.53)	3.42 (1.60)
Smoking	0.14 (0.61)	0.00 (0.00)
Exercise	2.33 (1.06)	2.18 (1.13)
Number of sputum samples	1.82 (2.17)	2.25 (2.89)

FEV1: Forced expiratory volume in 1 second, FVC: forced vital capacity, SEMCD: Self-Efficacy to Manage Chronic Disease Scale, SGRQ: St George’s Respiratory Questionnaire, LINQ: lung information needs questionnaire; BACI: Bronchiectasis Aetiology Comorbidity Index. Other than gender and smoking status, where data are represented as number and percentage, all data are represented as mean and standard deviation.

**Table 2.** Change in outcome measures from baseline to 12 months follow-up.

		Control		Intervention	Mean difference	p-value	Mean difference (adusted for baseline values). (Intervention – Control)	p-value
					(Intervention – Control)			
	n	Mean (SD)	n	Mean (SD)	Mean (95% CI)		Mean (95% CI)	
SEMCD	67	−0.2 (1.6)	60	−0.2 (1.4)	0.01 (−0.51,0.53)	0.96	0.14 (−0.37,0.64)	0.59
SGRQ								
Total	61	1.3 (11.7)	54	1.6 (11.5)	0.27 (−3.98,4.52)	0.9	0.24 (−4.01,4.49)	0.91
Activity	63	4.7 (17.8)	56	4.1 (14.3)	−0.60 (−6.48,5.27)	0.84	−0.73 (−6.49,5.02)	0.8
Impact	66	−1.0 (11.9)	59	0.1 (12.0)	1.21 (−2.95,5.37)	0.57	1.16 (−3.00,5.32)	0.59
Symptoms	68	0.6 (18.8)	60	−1.0 (21.2)	−1.54 (−8.48,5.39)	0.66	−1.47 (−8.03,5.09)	0.66
LINQ	57	12.18 (2.73)	49	11.45 (2.19)	−0.75 (-1.71,0.21)	0.124	−0.48 (−1.32,0.37)	0.265
Disease knowledge	50	−0.12 (0.92)	44	−0.14 (0.90)		0.894		
Medicines	47	−0.15 (0.62)	40	−0.30 (0.72)		0.303		
Self-management	47	−0.28 (1.36)	40	−0.10 (1.57)		0.854		
Smoking	52	0 (0.0)	42	0 (0.0)		NA		
Exercise	54	−0.07 (0.87)	44	0.18 (1.26)		0.238		
Sputum samples provided	93	1.48 (2.52)	95	2.09 (3.10)	1.54 (1.00, 2.35)	0.048	1.29 (0.88, 1.89)	0.197
								
Exacerbations					IRR		Adjusted IRR (95% CI)	
					(Intervention /control)			
					(95% CI)			
Exacerbations 0–12mths	34	3.6 (4.8)	21	6.3 (8.4)	1.64 (0.87,3.07)	0.12	1.57 (0.85, 2.87)	0.15
Hospital admissions or A&E attendances	84	0.73 (1.12)	87	1.07 (1.84)	1.36 (0.85, 2.18)	0.206	–	–

SEMCD: Self-Efficacy to Manage Chronic Disease Scale, SGRQ: St George’s Respiratory Questionnaire, LINQ: lung information needs questionnaire, A&E: accident and emergency. Intention to treat analysis. IRR: incidence rate ratio (intervention/control) ASD: Standard Deviation. CI: confidence interval. n: number with data available for analysis.

**Table 3.** Patients self-evaluation of their knowledge and confidence about bronchiectasis.

		Control		Intervention	
	Total number	Number (percentage)	Total number	Number (percentage	P
I do NOT feel confident in deciding when I need treatment	53	5 (9.4)	43	3 (7.0)	0.727*
I know which bacteria grows in my sputum/phlegm	48	18 (37.5)	41	15 (36.6)	0.929+
Sputum sample sent for testing…	50		41		0.111*
when I last had a flare up		25 (50)		28 (68.3)	
within the last 6 months		3 (6)		0 (0)	
within last 12 months		22 (44)		13 (31.7)	
Home supply of antibiotics	48		41		0.969*
I have a home supply & know when to use them		38 (79.2)		32 (78.1)	
I have a home supply but I don’t feel confident starting them		3 (6.3)		2 (4.9)	
I **don’t** have a home supply but would like to have some.		3 (6.3)		4 (9.8)	
I **don’t** have a home supply but I don’t want the responsibility		4 (8.3)		3 (7.3)	
**I feel confident** that I understand my condition, how to get it treated when necessary and explaining it to family or friends.	41	37 (90.2)	29	29 (96.6)	0.395*

The analysis was conducted by * Chi-squared test and + Fisher’s exact test.

Within the focus groups three participants out of eight had fully utilised the BET tool. Seven out of eight, felt the need for support with bronchiectasis, but not necessarily in the form of BET. Most participants of the focus group had already developed their own techniques for monitoring their condition. One of them said ‘*A lot of the things in there I already knew, but not everybody would, particularly the newly diagnosed wouldn’t’.* Another one said that

…what I would do is make it slightly simpler, I felt that sometimes I was repeating things. When you are filling it in, you are not well at the time and that makes it more difficult. I think that if someone could have reviewed my progress with me and guided me it might have been even more successful. 1105

However, those that did use BET reported having gained a clearer and better insight into the presentation and duration of their symptoms.

Without that [BET] I would have been lost. Because I was able to take the BET booklet with me to appointments and let them know what worked effectively and what wasn’t for instance when I went to the hospital I was able to say Meropenem and Tobramycin IVs to Dr R. 1056

The aspect that was mentioned most was the improved interaction and communication with healthcare professionals and secondly the self-care behaviours e.g. sputum testing and airway clearance. Emerging themes ranged from impact of the disease on social interactions; embarrassment, change of role and isolation, to the challenges of taking antibiotics influenced by side-effects, media messages and the complexities of intravenous self-administration (see appendix). An overarching theme was the need for informed guidance and support illustrated by the following extracts

From a personal basis not being able to pick up a phone and say to somebody do you think that it is alright? Do you think that I can do something to improve things? If you know someone who knows a lot about it that would be wonderful. A nurse to talk to. 1044

It was nice as I mentioned earlier to speak to a GP who was knowledgeable and knew exactly what I was saying. I do remember it was a yippee moment. But sadly that person is leaving. 1091

There is no easy flow of information or updates to patients, they get nothing]…[For a majority of my housebound patients they do not get regularly reviewed by either a GP or a hospital consultant. 2001

The intervention was estimated to be £40.11 ($52.95) per participant: 15 minutes per participant to arrange the phone calls, 24 minutes for the education sessions, £176 ($232.32) for staff training and £245 ($323.40) for printing BET. Table 4 summarises the mean QALY scores. The mean incremental cost was estimated for the intervention group, compared to the control group, to be £355.94 ($469.85) (95% confidence interval (CI) –£444.97 to £1156.85 (-$587.36 to $1527.04) and the mean QALY score to be 0.006 higher (95% CI –0.042 to 0.053). This resulted in an ICER of £64,223 ($84,774). According to the CEAC there was a 36.3% probability that the intervention was cost-effective at a λ of £20,000 ($26,400) per QALY.

**Table 4.** Quality adjusted life years score for intervention and control groups.

		Control		Intervention
	n	mean (SD)	n	mean (SD)
Baseline EQ-5D-3 L	103	0.709 (0.297)	101	0.716 (0.278)
3 month EQ5D-3 L	73	0.724 (0.285)	60	0.751 (0.251)
6 month EQ5D-3 L	58	0.704 (0.300)	48	0.701 (0.319)
9 month EQ5D-3 L	62	0.655 (0.323)	53	0.691 (0.319)
12 month EQ5D-3 L	65	0.737 (0.270)	58	0.689 (0.306)
QALY	63	0.723 (0.263)	57	0.709 (0.285)

There was no difference in the QALY score between the two groups. n=Number for whom data were available; SD=standard deviation; QALY=Quality Adjusted Life Years over 12 months.

## Discussion

We did not show that the use of BET had a beneficial effect in terms of self-efficacy, HRQOL or clinically relevant disease outcome measures such as exacerbations or hospitalisations or costs. The uptake into the study was high reflecting patients desire to be involved with and assist initiatives to increase their education and support for their condition. However, participants did not find the self-management tool to be valuable as, although the action plan was brief, overall BET was too onerous to complete and few participants used it. The participants did not feel more informed about their condition and there was no change in their behaviour. None of the participants were newly diagnosed and many had developed their own techniques to monitor and manage their disease. This was despite the involvement of patients with bronchiectasis in the development of BET although they were possibly self-selected in terms of their enthusiasm for the intervention.

Unfortunately the patient withdrawal was higher than we expected and therefore our study was underpowered. This may be due to the lack of study visits, and face-to-face contact with researchers, or to the burden of literacy represented by the intervention and patient reported outcome and cost measures. The low intensity nature of the study visits but relatively high questionnaire burden may have resulted in disengagement with the study. Also the BET tool was not evaluated within a larger process of care and it could not be modified by the clinical team or patient. It is likely that if the healthcare professionals involved had been regularly reviewing and updating the action plan or educational material or notepads contained within BET, it would have been used more. Although the separate elements of a care bundle need to be individually assessed,^35^ action plans are more effective if integrated within healthcare^36^; and lack of review of asthma self-management plans by healthcare professionals leads to lack of interest by patients.^37^

The action plan in BET was accompanied by brief written and one-to-one patient education as we envisaged that would be the case in clinical practice. This was delivered by phone as this was more convenient, permitted standardised training throughout a multi-centre study and was preferred by the patients. Many people in the focus groups liked the telephone education and indeed structured telephone support has been shown to be beneficial for people with chronic heart failure.^38^ However, a more intensive programme or one integrated within the practice and championed by healthcare providers may have had greater uptake and benefit.^39^ We did not include training on skills such as problem solving, decision-making, goal setting and emotional management. Diabetes standards suggest greater than 10 hours of support are required for implementation of self-management plans.^40^

We had broad inclusion criteria for this study, only requiring documented evidence of diagnosis and one exacerbation in the previous year, to maximise generalizability. However our participants had less impaired HRQOL compared to other trials^41^ (but similar to observational studies^16^) and the majority of individuals felt confident about bronchiectasis in both groups at the end of the study. It is possible that the reason for lack of detectable benefit is that the patients had relatively mild disease of long duration (average more than a decade) and had already developed mechanisms for managing their disease so did not benefit from this alternative tool. Indeed, it was suggested in the focus groups that individuals with newly diagnosed disease would find the tool more beneficial but we did not purposively sample those with a good response for the focus groups

## Conclusion

We have shown that BET did not improve outcomes. Many participants had mild disease, already developed self-management techniques and/or considered themselves confident with their condition. The telephone education was appreciated by participants and could be utilised to a greater extent in the future. BET should not be used as it stands but a simplified version should be evaluated in newly diagnosed patients, probably in the context of a wider care package with more intensive support. Recruitment into the study was high suggesting a clinical need but future studies should allow for up to 50% withdrawal rate or utilise less burdensome outcome measures, perhaps capturing patients ability to communicate with healthcare professionals or bronchiectasis specific HRQOL.

## Supplemental Material

Supplemental Material, sj-docx-1-crd-10.1177_1479973120948077 - Randomised controlled trial of the effect, cost and acceptability of a bronchiectasis self-management intervention Click here for additional data file. Supplemental Material, sj-docx-1-crd-10.1177_1479973120948077 for Randomised controlled trial of the effect, cost and acceptability of a bronchiectasis self-management intervention by Claire Brockwell, Andrea Stockl, Allan Clark, Garry Barton, Mark Pasteur, Robert Fleetcroft, Janice Hill and Andrew M Wilson in Chronic Respiratory Disease

Supplemental Material, sj-docx-2-crd-10.1177_1479973120948077 - Randomised controlled trial of the effect, cost and acceptability of a bronchiectasis self-management intervention Click here for additional data file. Supplemental Material, sj-docx-2-crd-10.1177_1479973120948077 for Randomised controlled trial of the effect, cost and acceptability of a bronchiectasis self-management intervention by Claire Brockwell, Andrea Stockl, Allan Clark, Garry Barton, Mark Pasteur, Robert Fleetcroft, Janice Hill and Andrew M Wilson in Chronic Respiratory Disease

Supplemental Material, sj-docx-3-crd-10.1177_1479973120948077 - Randomised controlled trial of the effect, cost and acceptability of a bronchiectasis self-management intervention Click here for additional data file. Supplemental Material, sj-docx-3-crd-10.1177_1479973120948077 for Randomised controlled trial of the effect, cost and acceptability of a bronchiectasis self-management intervention by Claire Brockwell, Andrea Stockl, Allan Clark, Garry Barton, Mark Pasteur, Robert Fleetcroft, Janice Hill and Andrew M Wilson in Chronic Respiratory Disease

Supplemental Material, sj-docx-4-crd-10.1177_1479973120948077 - Randomised controlled trial of the effect, cost and acceptability of a bronchiectasis self-management intervention Click here for additional data file. Supplemental Material, sj-docx-4-crd-10.1177_1479973120948077 for Randomised controlled trial of the effect, cost and acceptability of a bronchiectasis self-management intervention by Claire Brockwell, Andrea Stockl, Allan Clark, Garry Barton, Mark Pasteur, Robert Fleetcroft, Janice Hill and Andrew M Wilson in Chronic Respiratory Disease

## Abbreviations

BET: Bronchiectasis Empowerment Tool
CEAC: Cost effectiveness acceptability ratio
CI: Confidence Interval
EQ-5D-3L: EuroQol-5D 3 level version
FEV1: Forced Expiratory Value in one second
GBP: Great British Pound
HRCT: High resolution computed tomography
ICER: Incremental cost-effectiveness ratio
ISRCTN: International Standard Randomised Controlled Trials Number
LINQ: Lung Information Needs Questionnaire
λ: Maximum acceptable ratio relating to CEAC
n: Number with data available
NHS: National Health System
NICE: National Institute for Health and Care Excellence
p: Probability value
QALY: Quality Adjusted Life Year
SGRQ: St George’s Respiratory Questionnaire
SEMCD: Self-Efficacy to Manage Chronic Disease Scale
SD: Standard Deviation
