# Supplementary figures and images for: Randomised controlled trial of the effect, cost and acceptability of a bronchiectasis self-management intervention
Source: Chron Respir Dis. 2020 Dec 2;17:1479973120948077. doi: 10.1177/1479973120948077 (PMC7716069; doi:10.1177/1479973120948077)

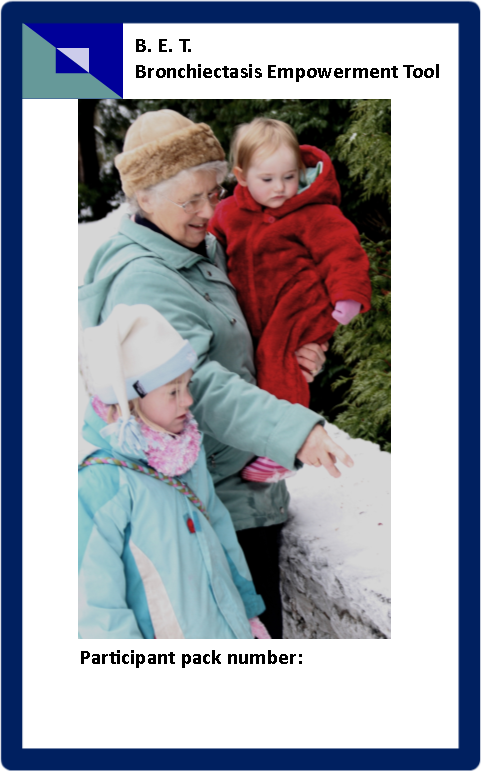


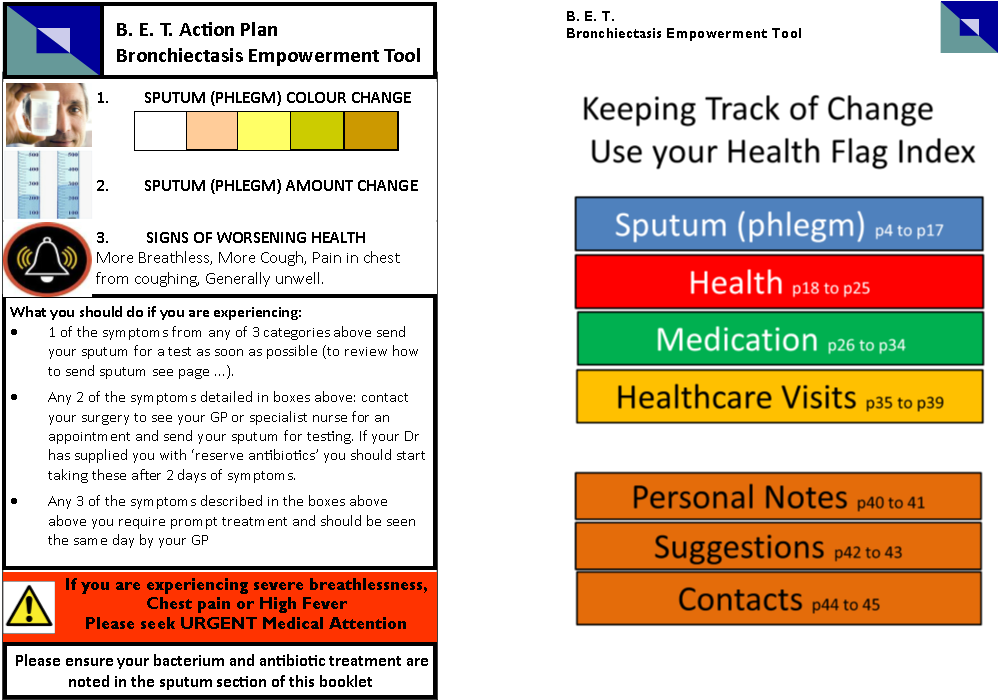


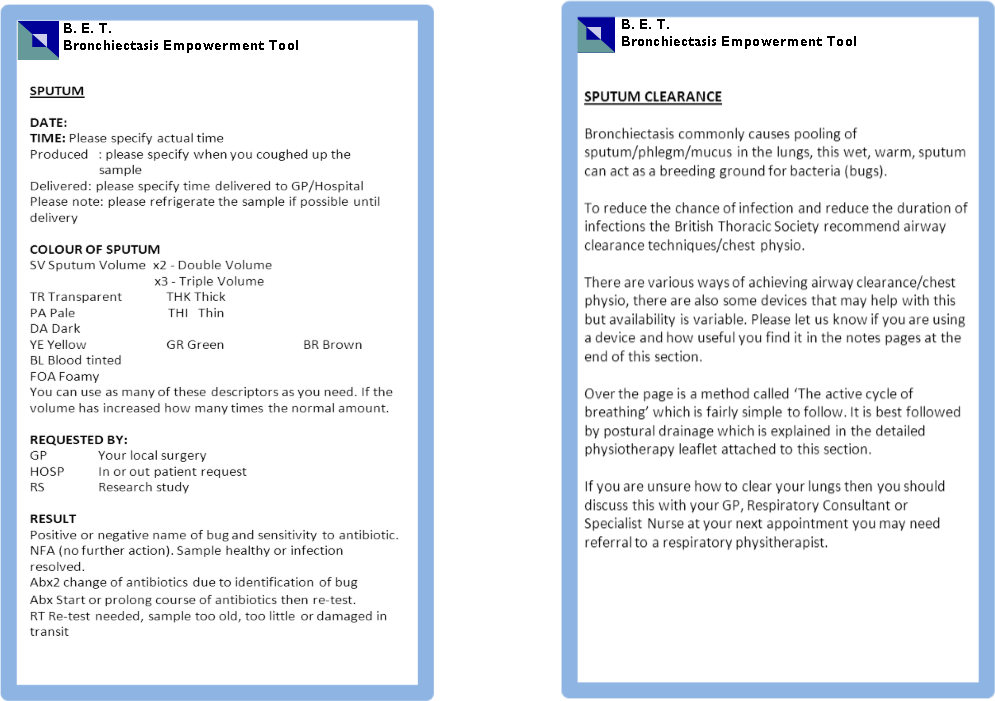


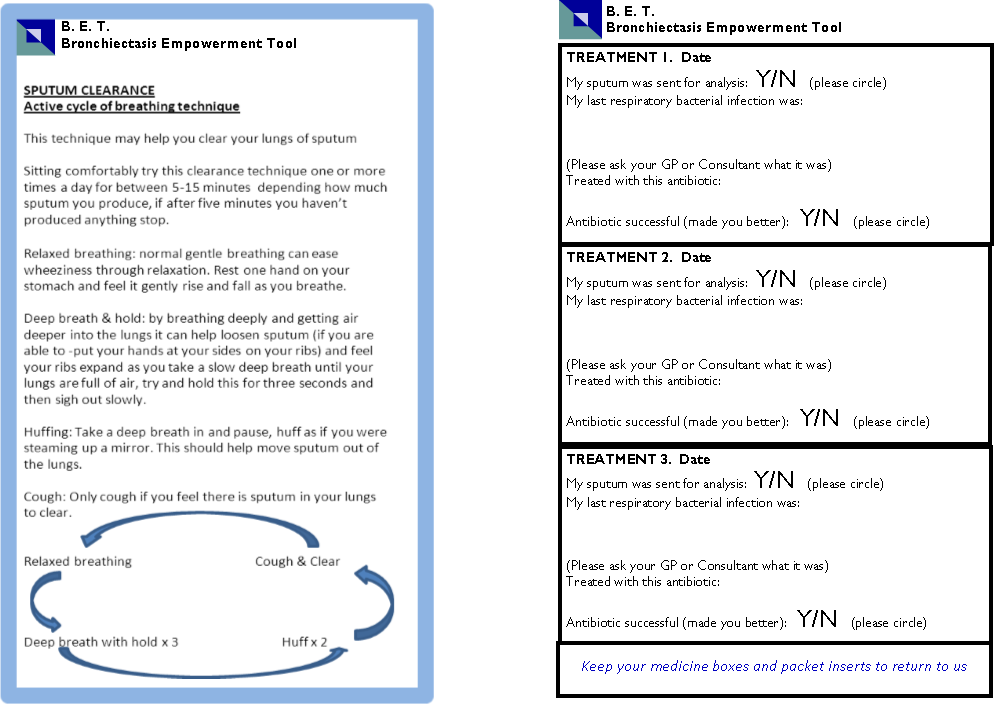


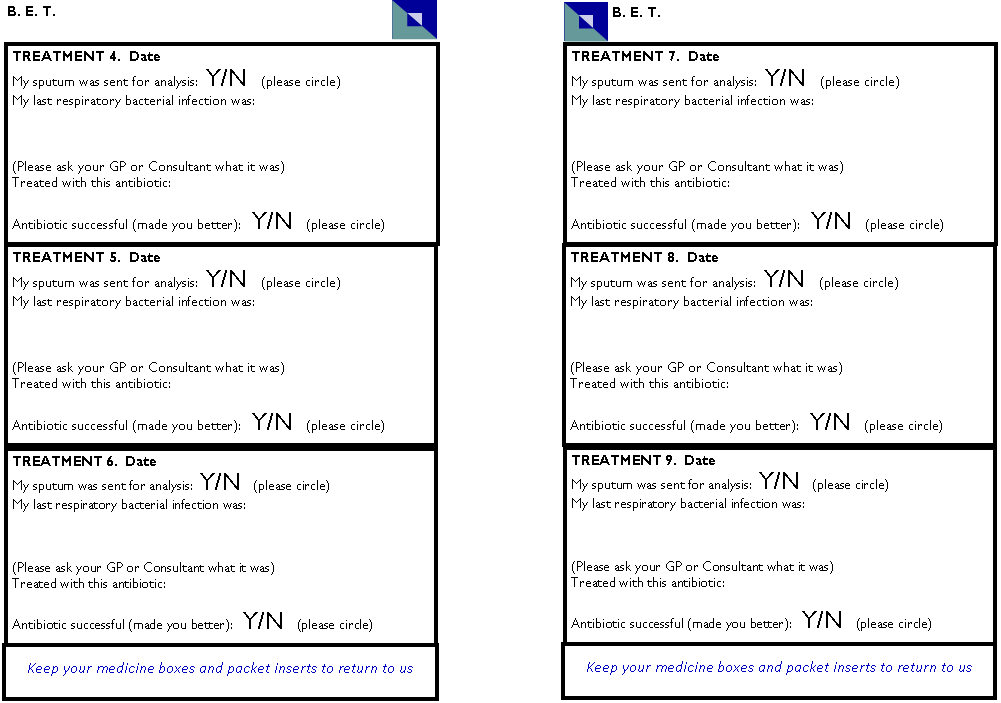


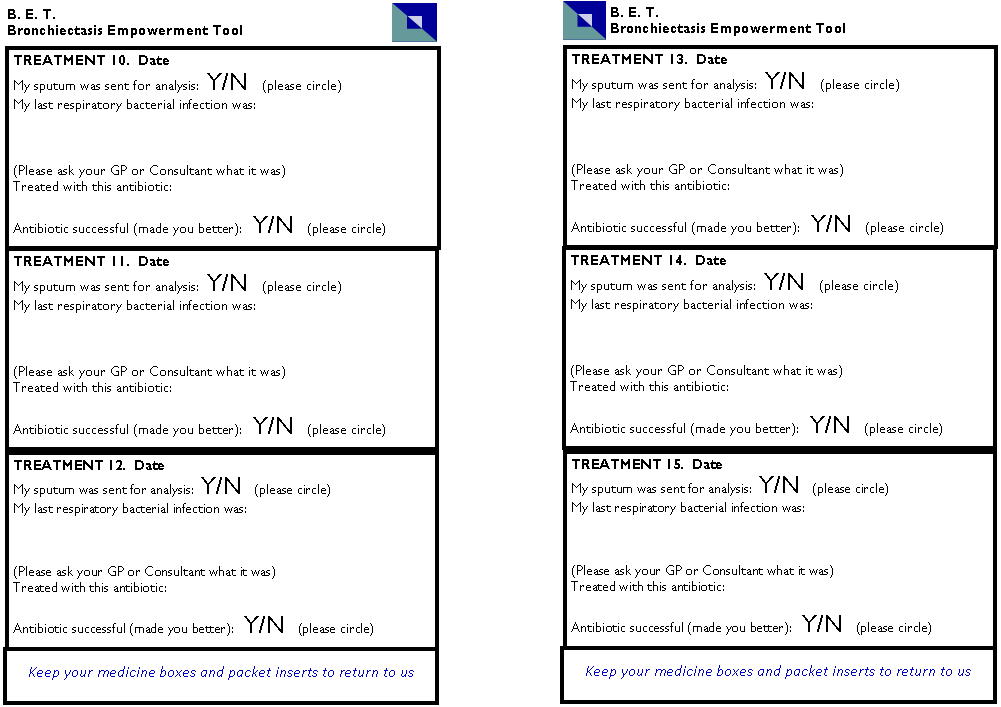


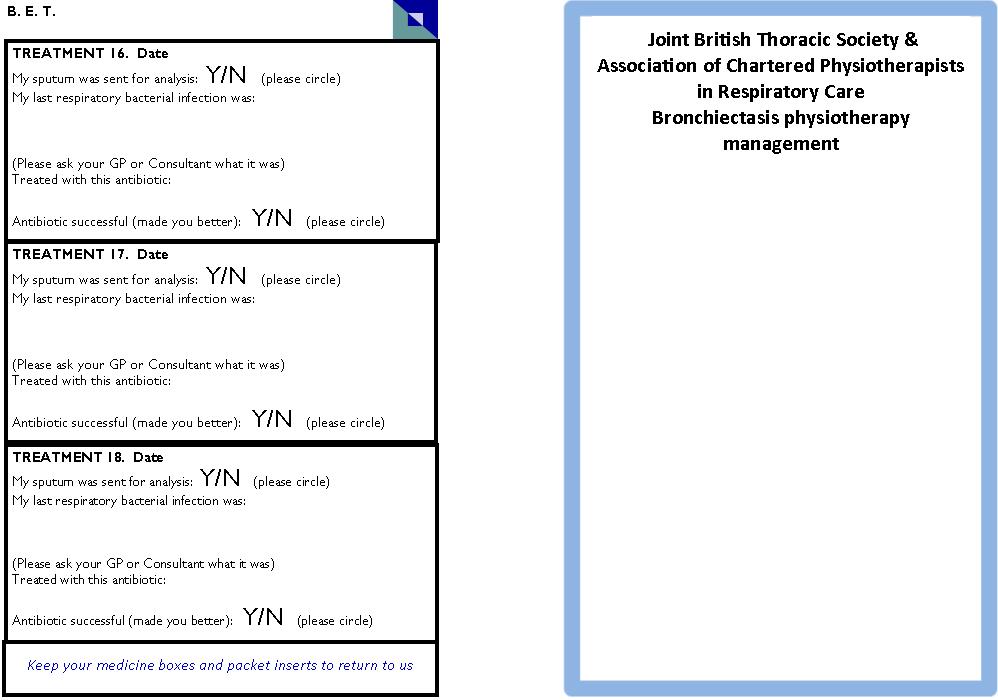


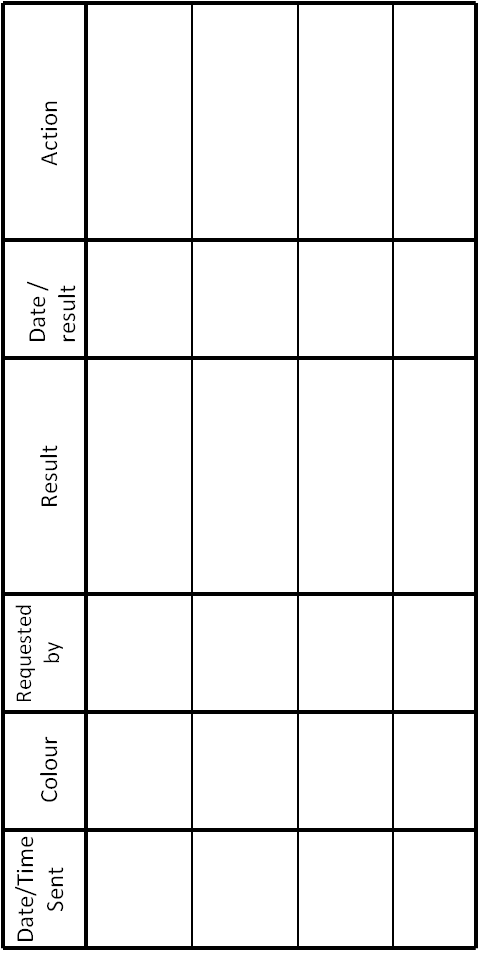

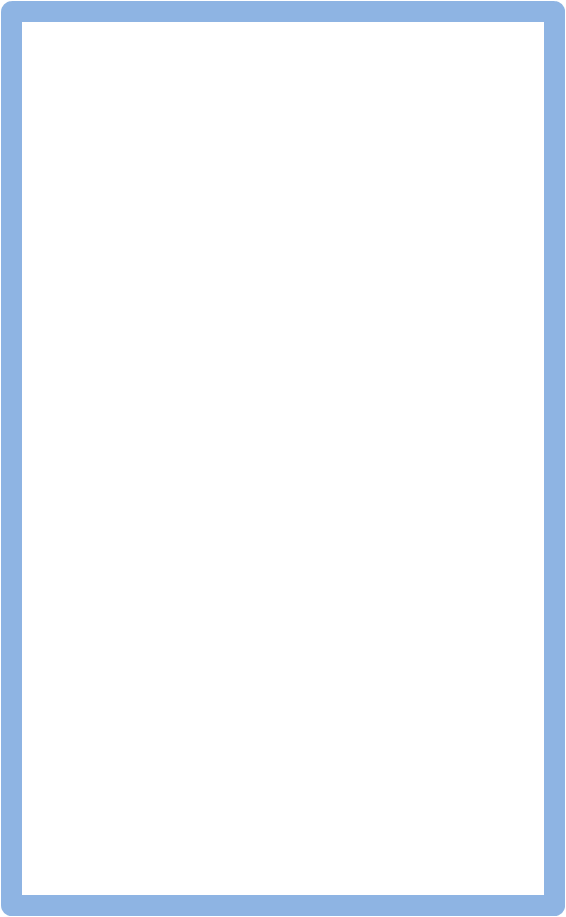

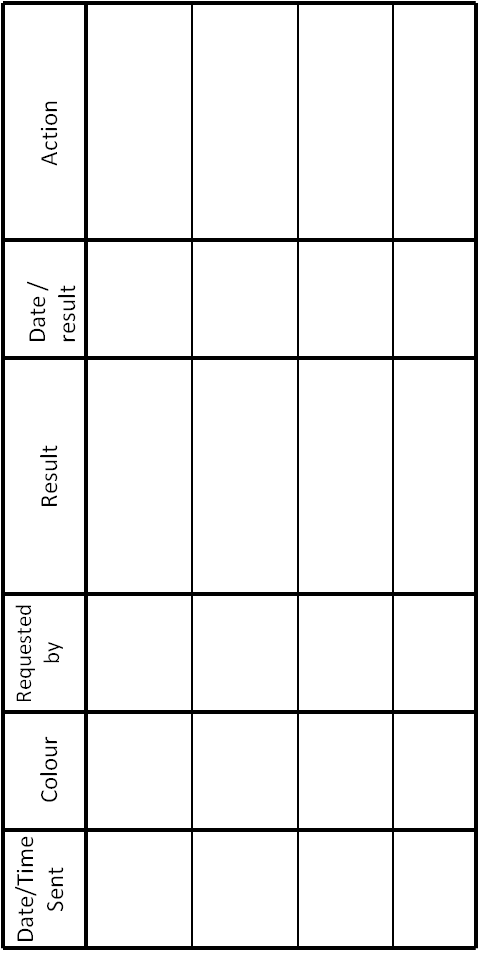

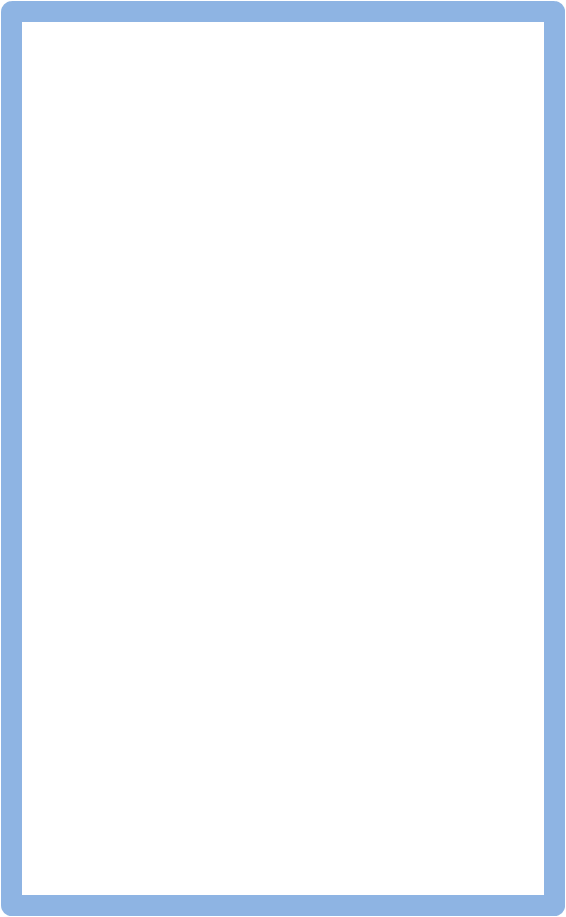


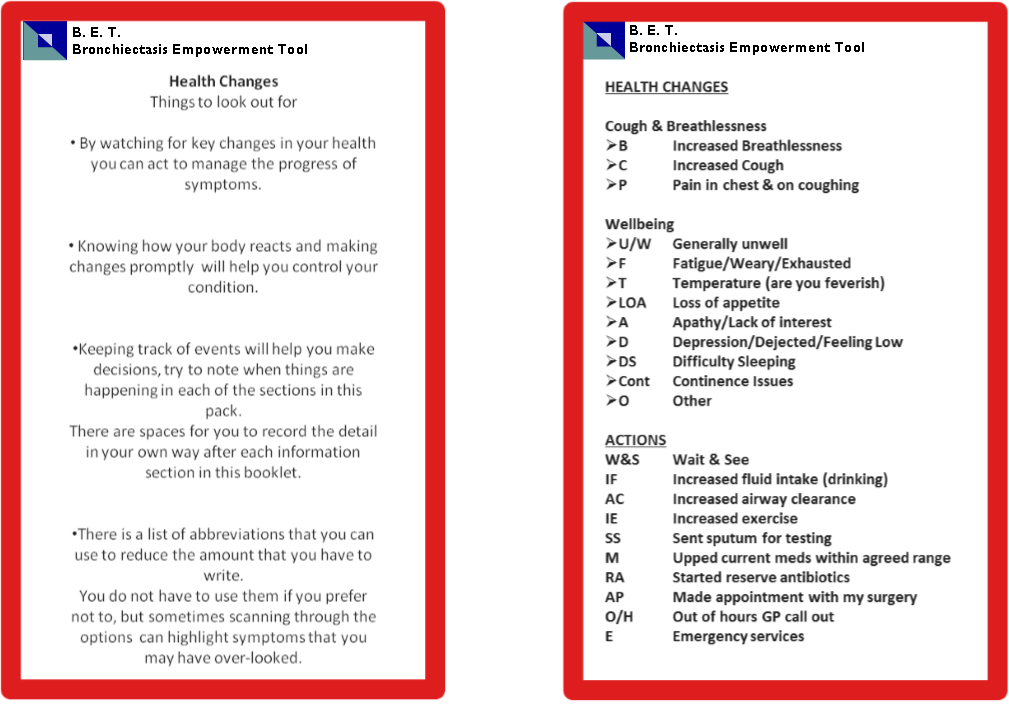


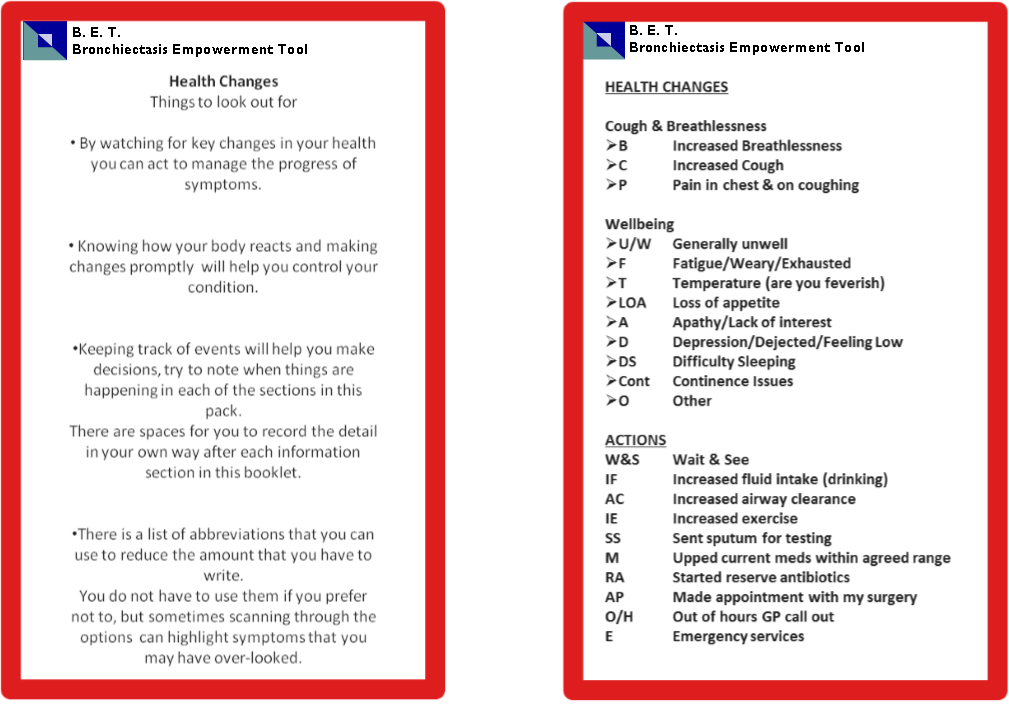


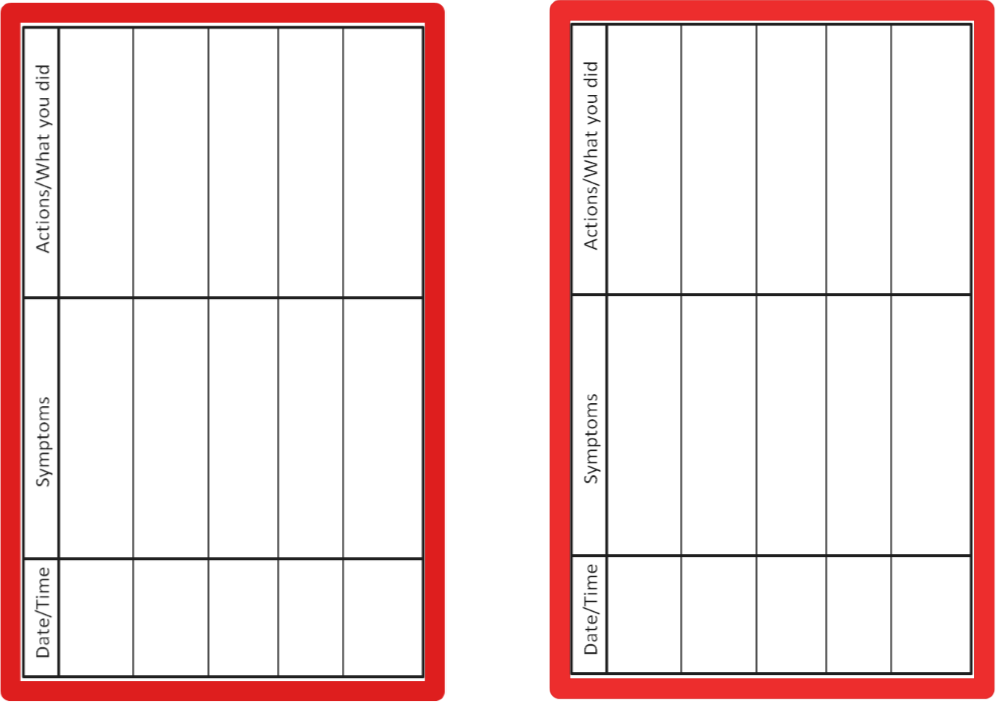


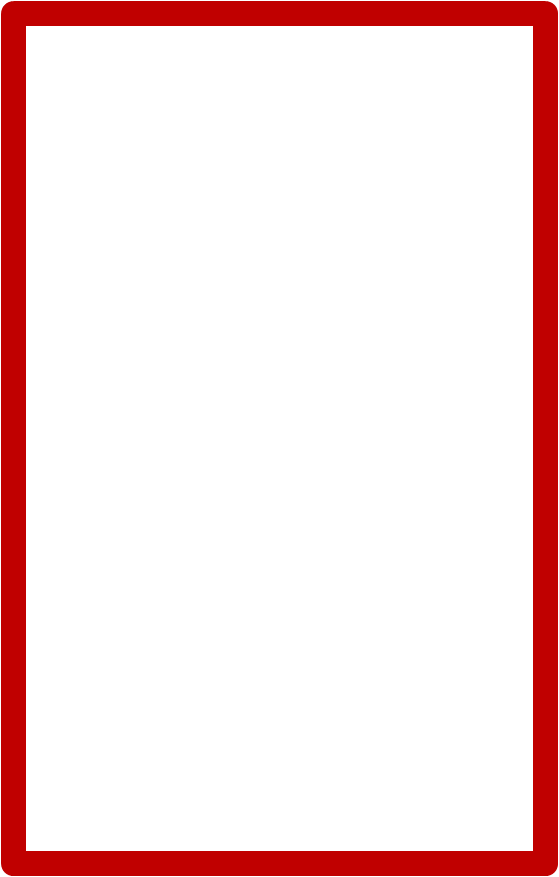

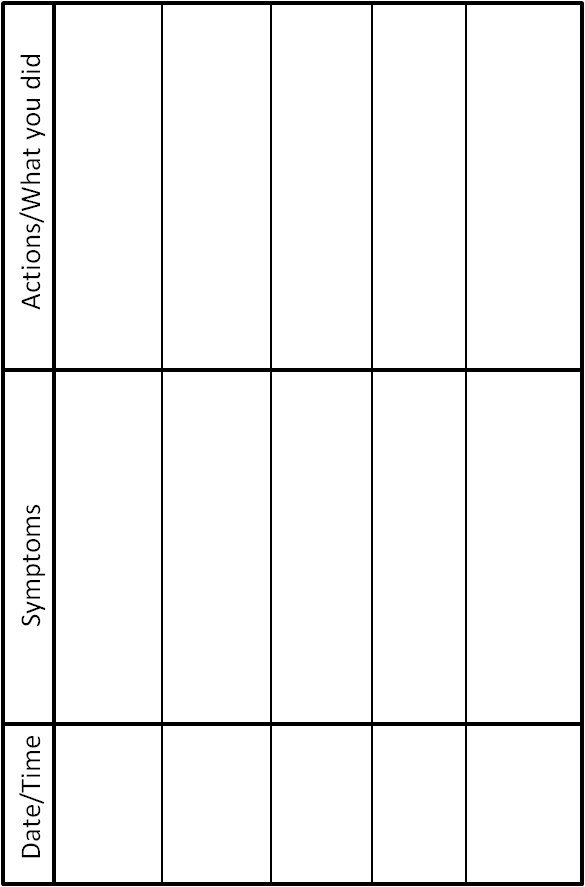

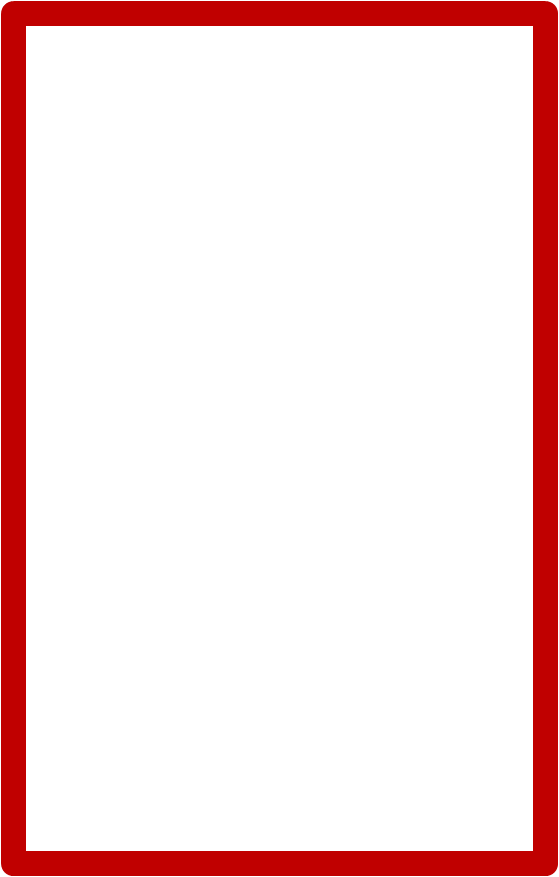

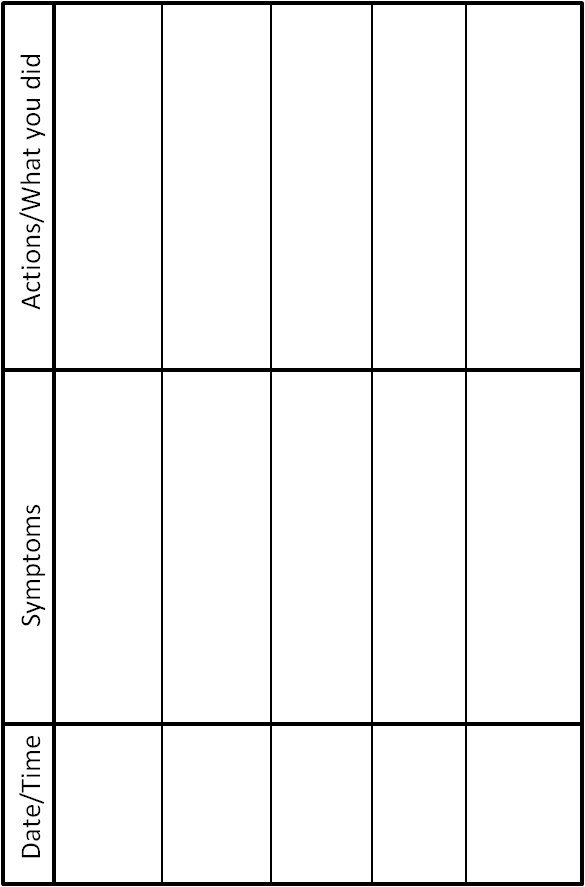


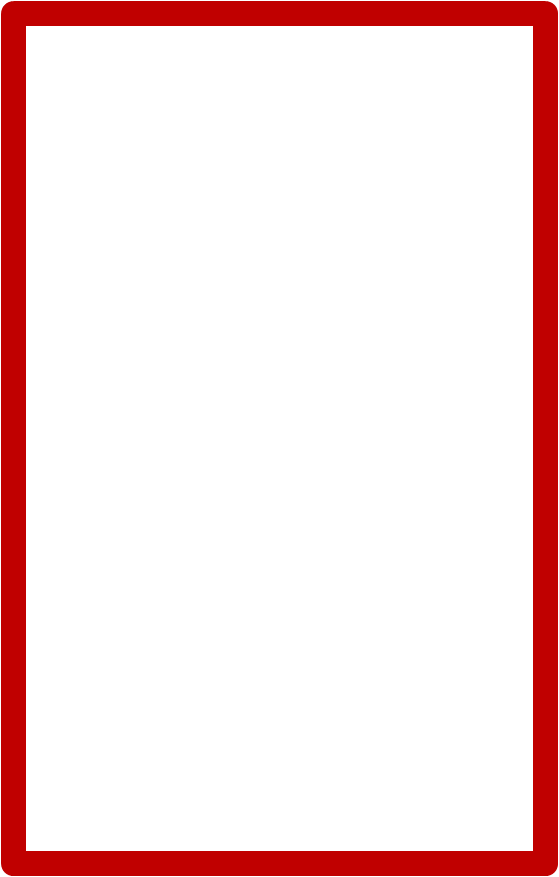

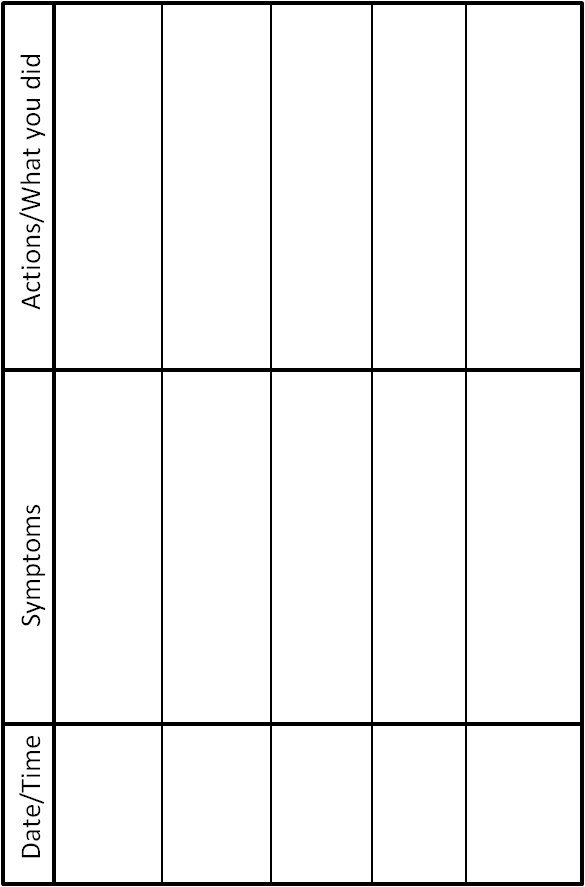

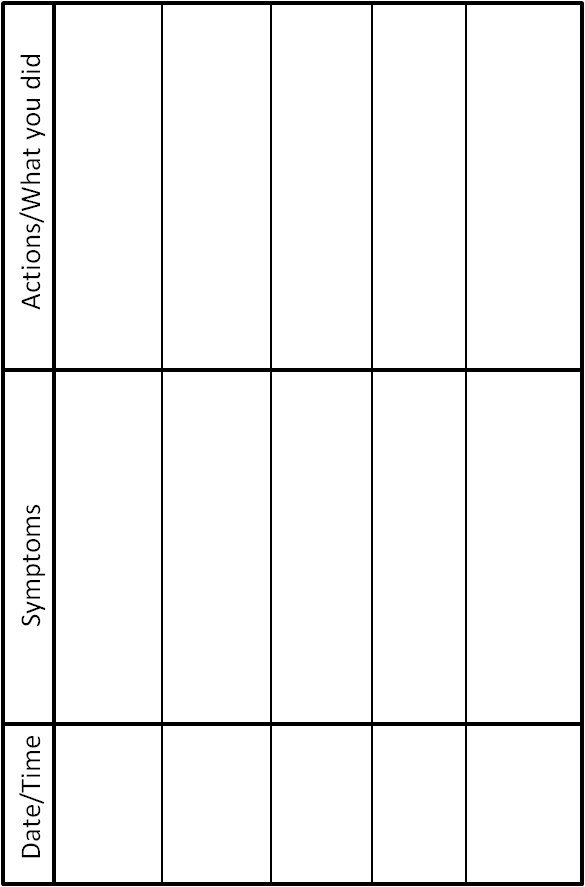

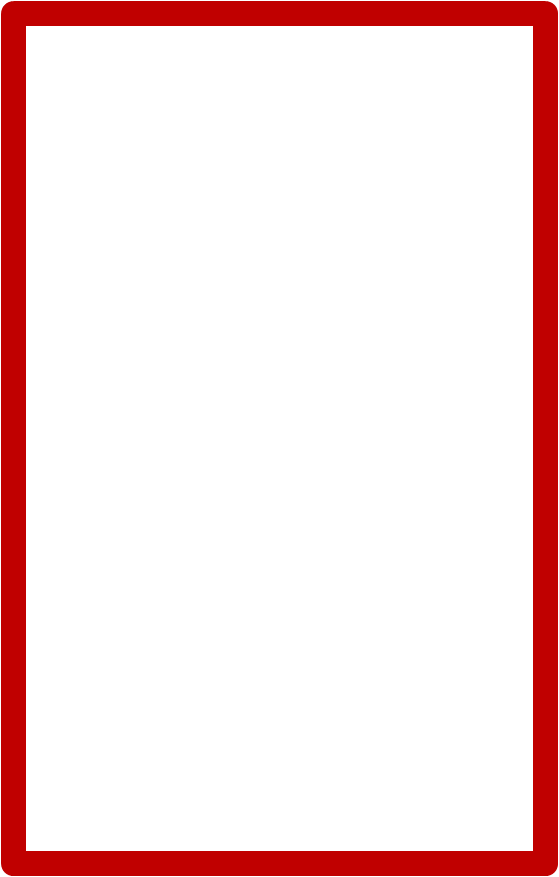


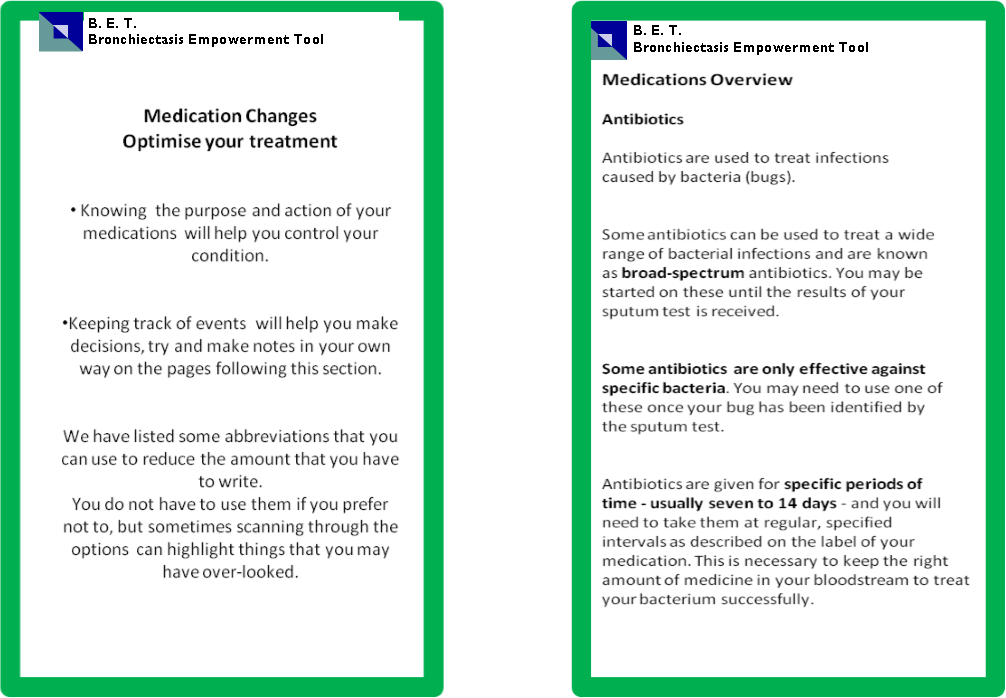


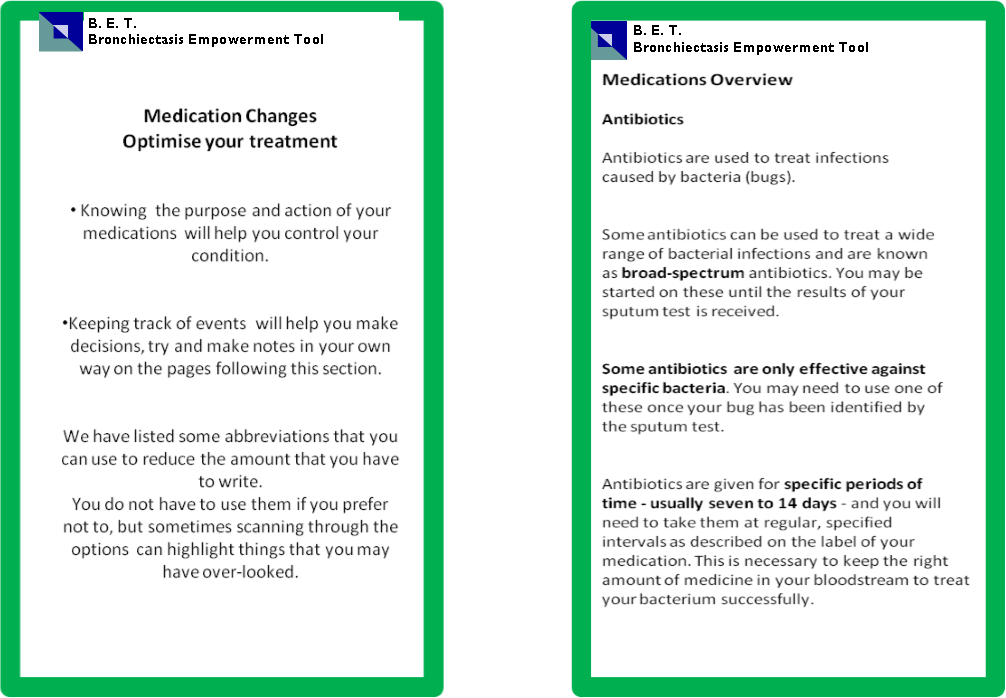


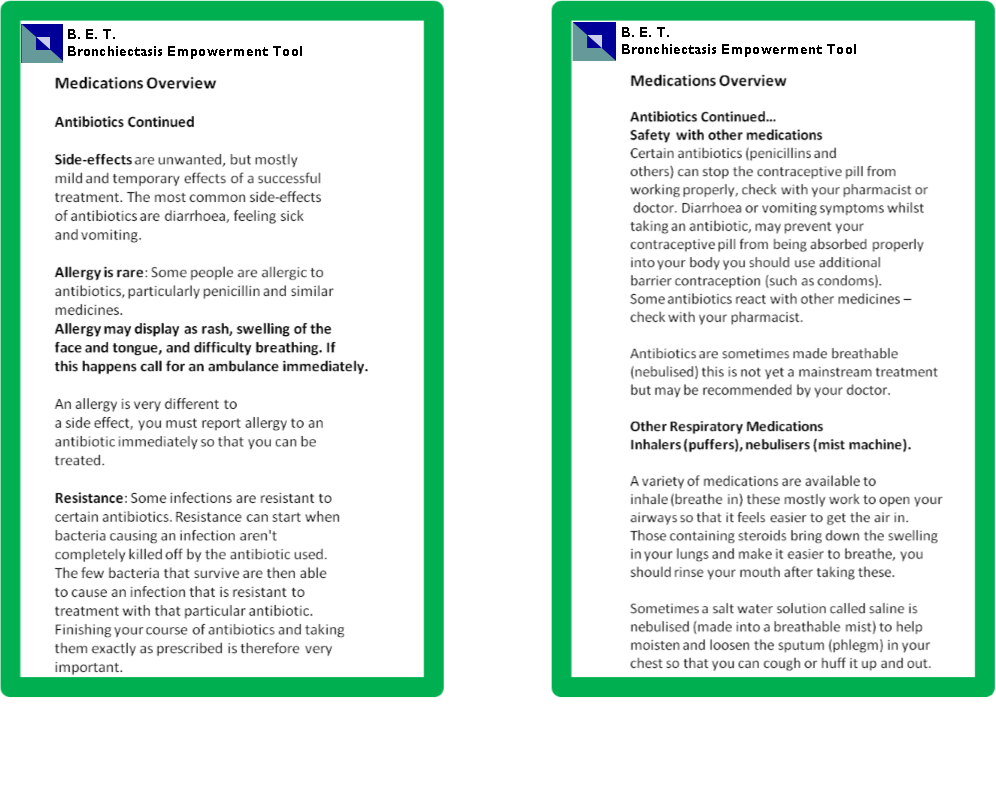

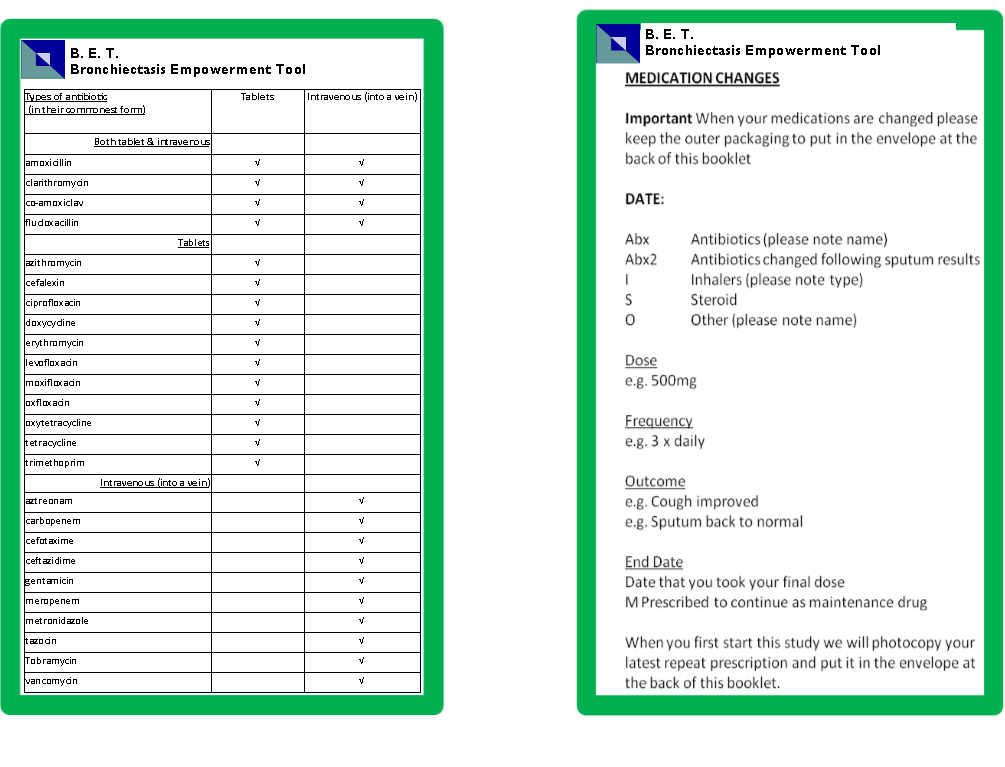


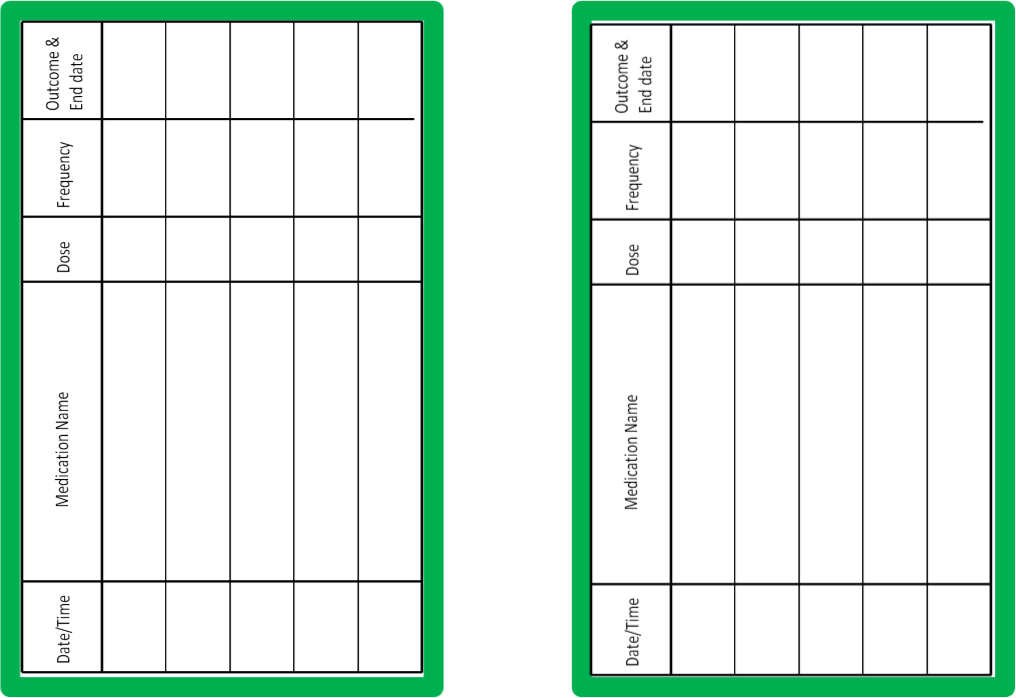


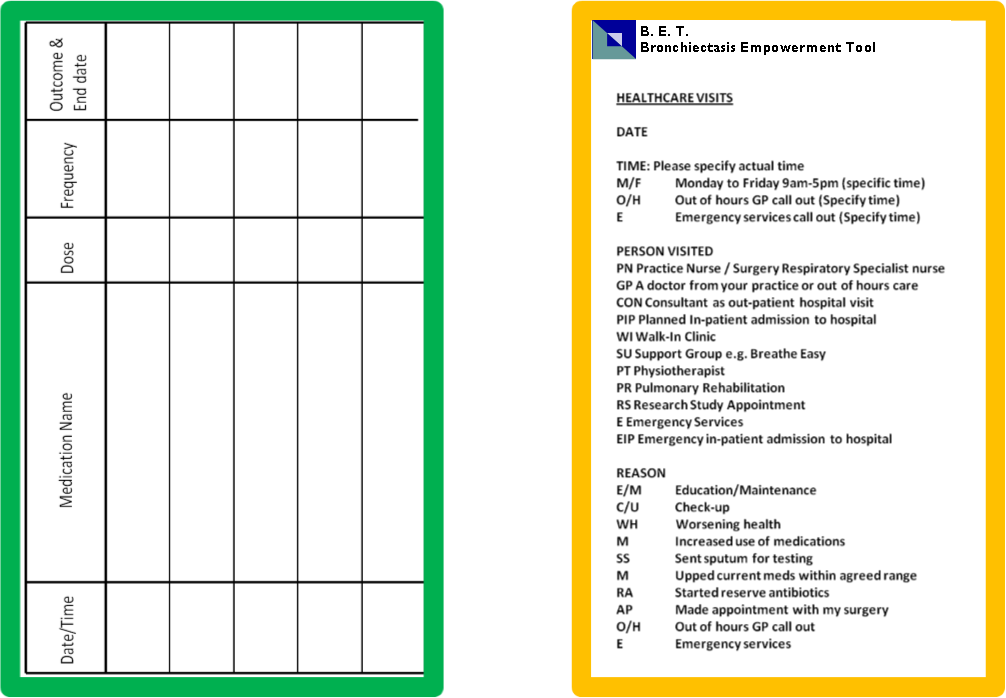


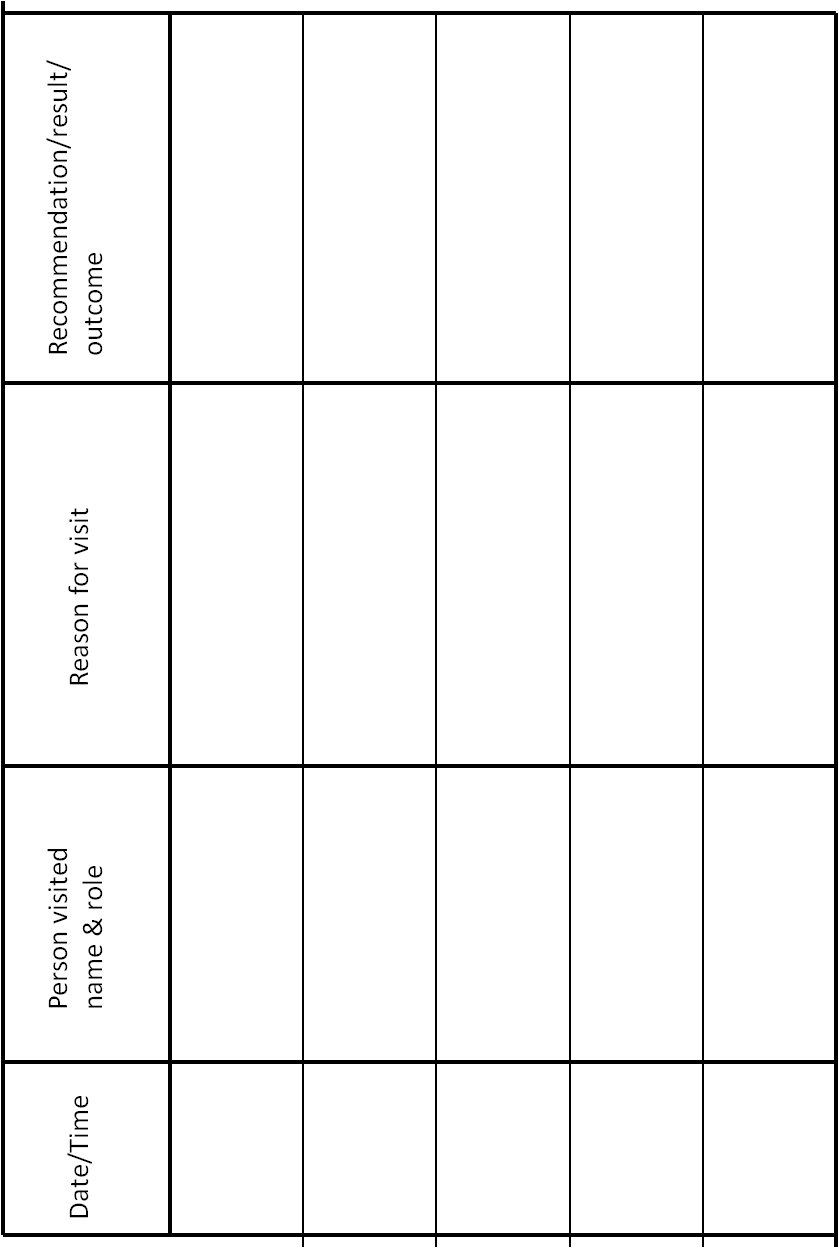

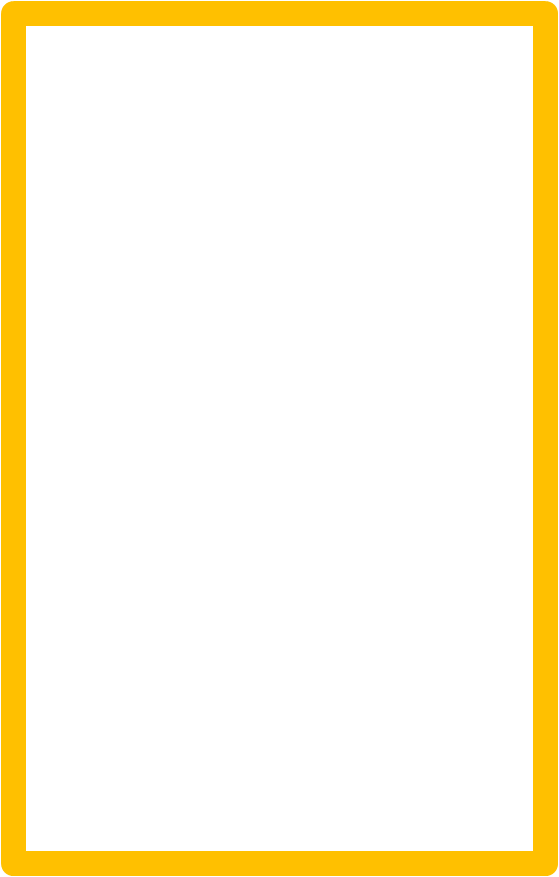

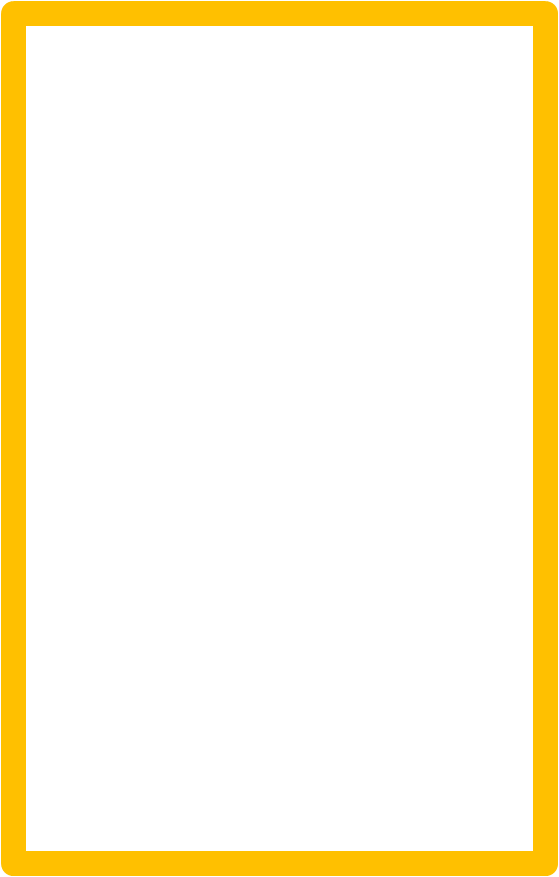

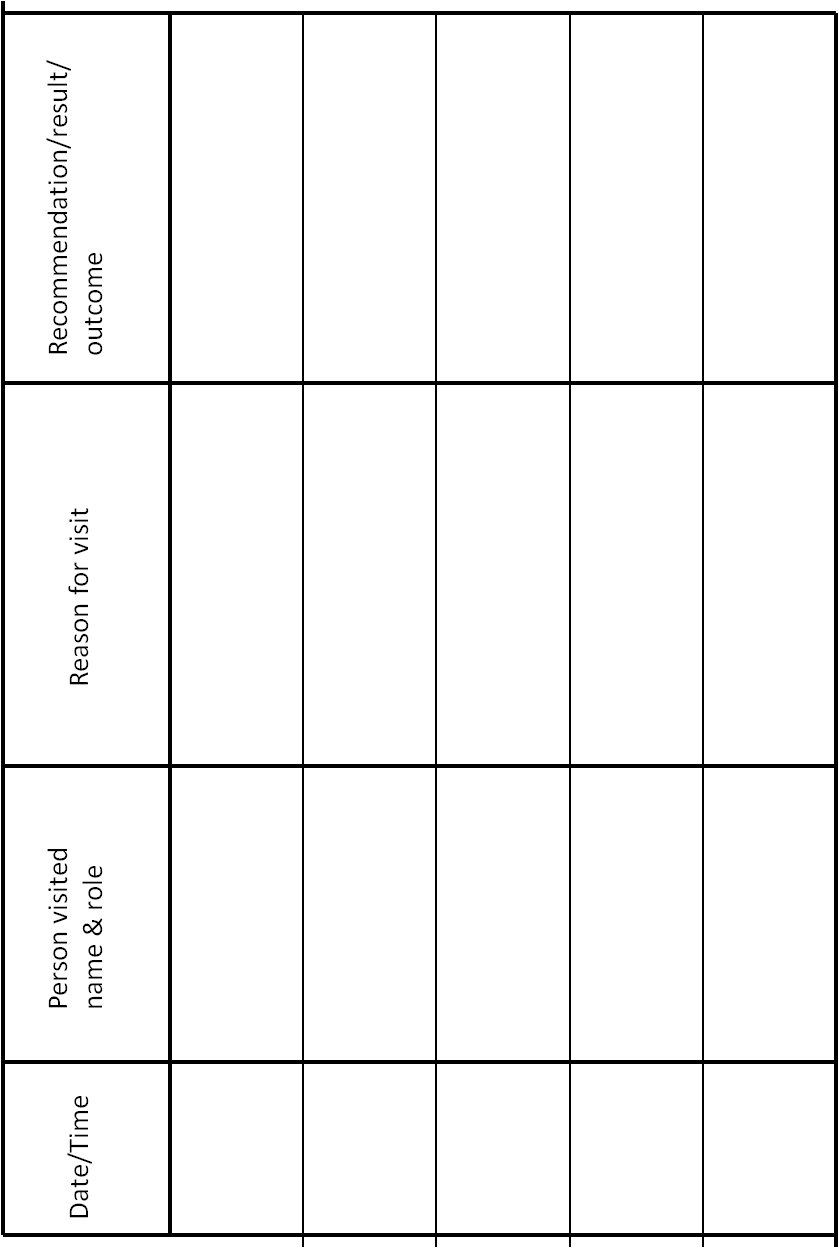


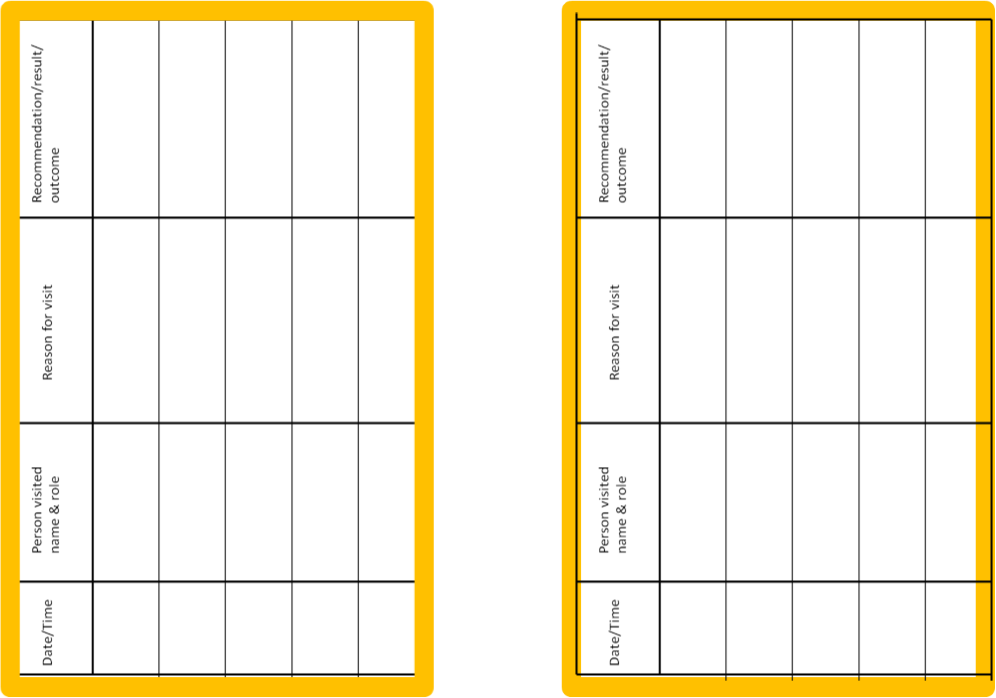

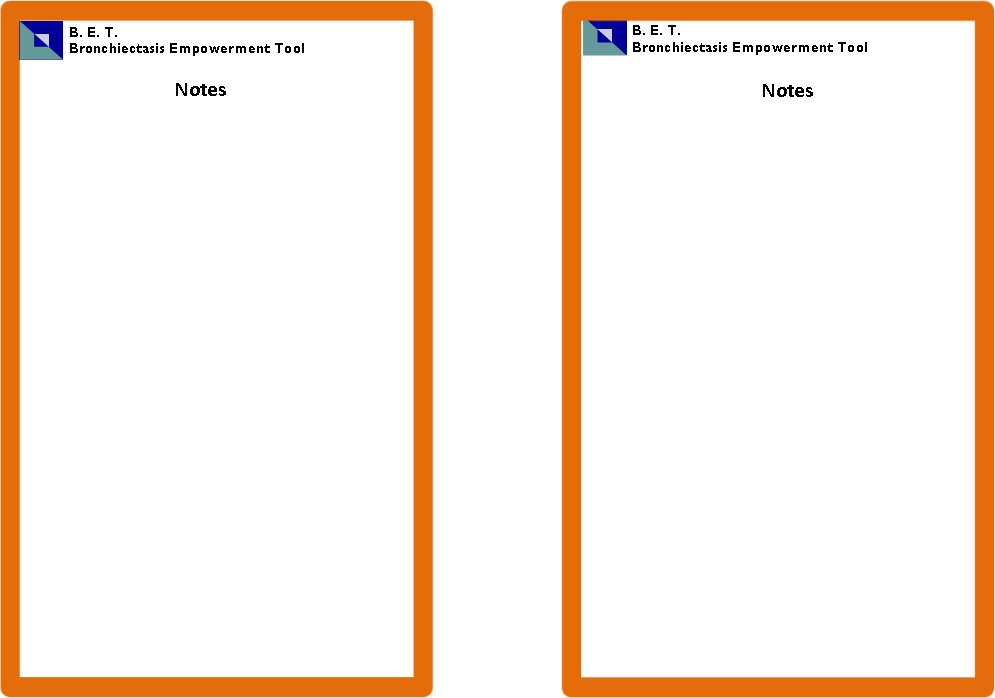


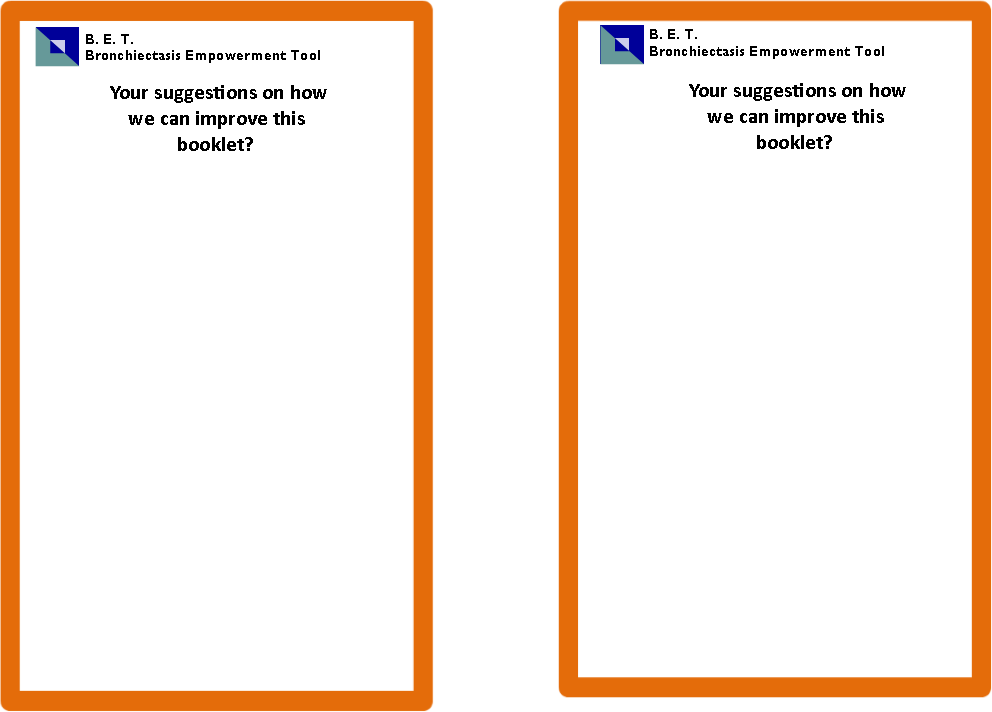


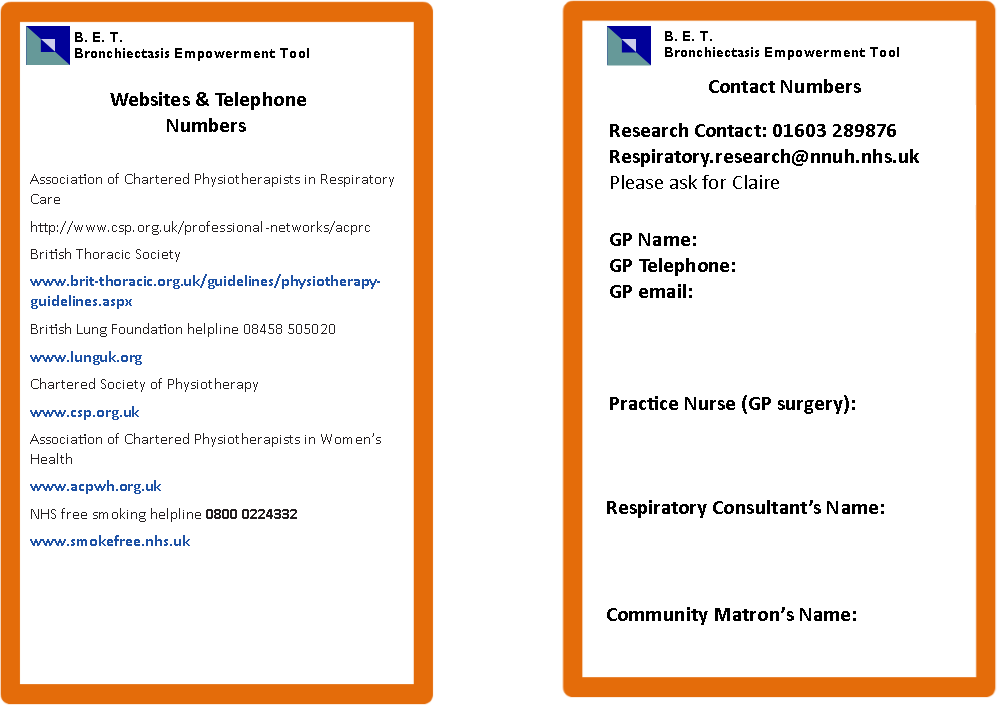


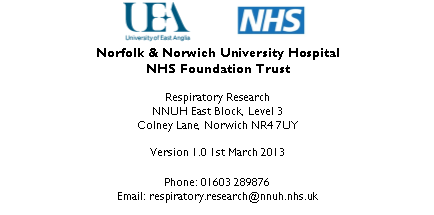

Supplement: Supplemental Material, sj-docx-1-crd-10.1177_1479973120948077 - Randomised controlled trial of the effect, cost and acceptability of a bronchiectasis self-management intervention [file sj-docx-1-crd-10.1177_1479973120948077.docx]

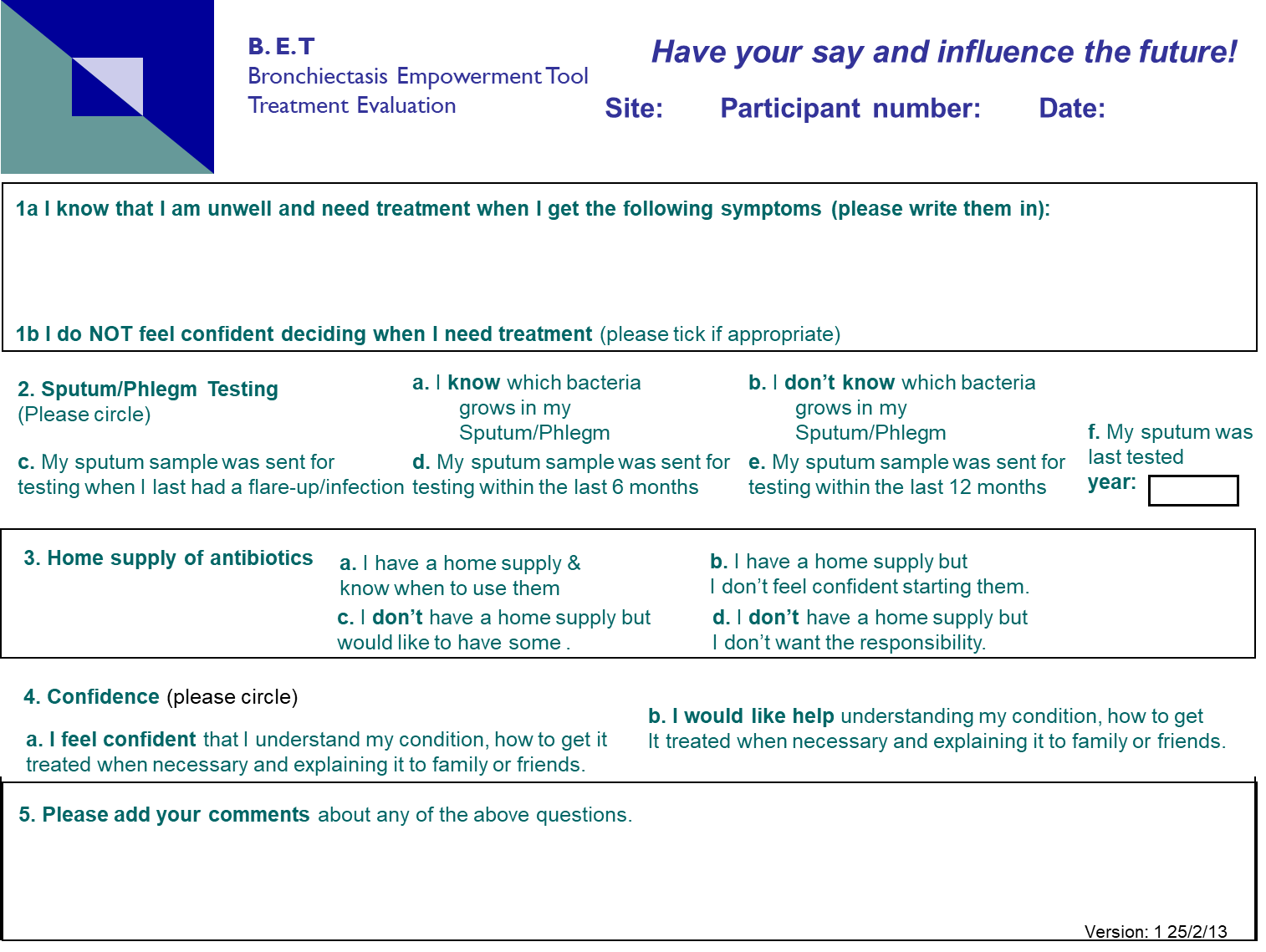

Supplement: Supplemental Material, sj-docx-2-crd-10.1177_1479973120948077 - Randomised controlled trial of the effect, cost and acceptability of a bronchiectasis self-management intervention [file sj-docx-2-crd-10.1177_1479973120948077.docx]
