# Supplementary material for: Randomised controlled trial of the effect, cost and acceptability of a bronchiectasis self-management intervention
Source: Chron Respir Dis. 2020 Dec 2;17:1479973120948077. doi: 10.1177/1479973120948077 (PMC7716069; doi:10.1177/1479973120948077)
Supplement: Supplemental Material, sj-docx-4-crd-10.1177_1479973120948077 - Randomised controlled trial of the effect, cost and acceptability of a bronchiectasis self-management intervention [file sj-docx-4-crd-10.1177_1479973120948077.docx]

| **Intervention** | **Time (months)** | | | | |
| --- | --- | --- | --- | --- | --- |
|  | 0 | 3 | 6 | 9 | 12 |
| Consent | X |  |  |  |  |
| Demographics | X |  |  |  |  |
| Drug history | X | X | X | X | X |
| Self-Efficacy Score | X | X | X | X | X |
| SGRQ | X | X | X | X | X |
| EuroQol 5-dimension | X | X | X | X | X |
| LINQ | X |  |  |  |  |
| Exacerbations | X | X | X | X | X |
| Socioeconomic status | X |  |  |  |  |
| Non validated questionnaire |  |  |  |  | X |
| Healthcare Utilisation | X | X | X | X | X |
| Focus Groups |  |  |  |  | X |
