# Supplementary material for: Randomised controlled trial of the effect, cost and acceptability of a bronchiectasis self-management intervention
Source: Chron Respir Dis. 2020 Dec 2;17:1479973120948077. doi: 10.1177/1479973120948077 (PMC7716069; doi:10.1177/1479973120948077)
Supplement: Supplementary file 3 [file sj-docx-3-crd-10.1177_1479973120948077.docx]

Qualitative evaluation of BET from a patient and health care professional point of view

Evaluation of predictors or factors of response to BET

Predictors of response	Participants
	Difficulty getting healthcare appointments hospital or GP appointments (lack of GPs)
	Fear of Antibiotics (media driven)
	Living alone, lack of support when required, lack of comparison with others
	Severity of disease (difficulty keeping track when too ill/exhausted even to cough)
	Cognitive/health literacy issues with completing forms
	Sputum results are not always routinely given to the patient
	Those who were well forgot how to use the BET booklet by the time they were poorly enough to need it
	Follow-up at one or six months would be useful to ensure comprehension

FOCUS Group themes

	Themes	Patients	Secondary care Health Professional	Primary Care Health Professional
1	Access to specialist knowledge	Access to specialist knowledge		Access to specialist knowledge
2	Access to training and education	Access to training and education	Access to training and education. Self-motivated/time pressure/no standardised curriculum	Access to training and education. Self-motivated/time pressure/no standardised curriculum
3	Unaware/ease of access to network of services available	Unaware/ease of access to network of services available	Unaware/ease of access to network of services available	Unaware/ease of access to network of services available
4	Antibiotics	Fear of use and side-effects. Also fear of future resistance.	Advice from secondary care may be lost in translation and not materialise in primary care	Constant challenge to reduce use
5	Recognition of exacerbation	Recognition of exacerbation		Recognition of exacerbation
6	Understanding sputum results	Understanding sputum results		Understanding sputum results and impact on prescribing
7	Keeping track and recording disease	Keeping track and recording disease. Individualistic. Cognition/Health literacy/Personal preference		
8	Intravenous Antibiotics in hospital	No beds. Fear of other infections. Lack of control. Interrupts life. Difficulty getting sleep. Timely medication is a challenge	Lack of bed space. Lack of specialist support service	N/A (see title)
9	Intravenous antibiotics at home	Lack of adequately staffed specialist support service	Lack of funding/staff time for specialist support service	Lack of funding/staff time for specialist support service
10	Airway clearance training and other physiotherapy needs (also pelvic floor due to cough)	Airway clearance training and other physiotherapy needs (also pelvic floor due to cough). Often only trained when first diagnosed or acutely ill in hospital - annual refresher needed	Only enough physiotherapy staff hours for acute care and newly diagnosed	Physiotherapy availability difficult
11	Issues of communication and contradiction between primary and secondary care	Issues of communication and contradiction between primary and secondary care	Issues of communication and contradiction between primary and secondary care	Issues of communication and contradiction between primary and secondary care
12	Support and advice	Obtaining support and advice	Staffing levels do not provide the support & advice patients want	Staffing levels do not provide the support & advice patients want
13	Isolation	Isolation		Community staff recognised issues of isolation for people with bronchiectasis when they are unable to leave the home
14	Lack of peers or publicity (bronchiectasis relatively unknown)	Lack of peers or publicity (bronchiectasis relatively unknown)		
15	Social Impact. Embarrassment , change of role, lack of ability to plan ahead/attend events	Social Impact. Embarrassment , change of role, lack of ability to plan ahead/attend events		Community staff observed change in role and social impact for participants
16	Having the BET document helped improve knowledge and improved patient	Having the BET document helped improve knowledge and improved patient confidence in requesting care		
